# The effect of laser pulse on nonlinear thermoelasticity using an advanced analytical method

**DOI:** 10.1038/s41598-026-52771-6

**Published:** 2026-05-19

**Authors:** Wafaa B. Rabie, Hamdy M. Ahmed, Mohamed F. Ismail, Ayman Al-Ahwal, M. Elsaid Ramadan, Nesreen Sirelkhtam Elmki Abdalla, Mohammed H. Ali

**Affiliations:** 1 Department of Mathematics, Faculty of Science, Luxor University, Taiba, Luxor, Egypt; 2 Faculty of Computing, Information Technology, and Artificial Intelligence, Luxor National University, Luxor, Egypt; 3 Department of Physics and Engineering Mathematics, Higher Institute of Engineering El Shorouk Academy, Cairo, Egypt; 4 Faculty of Computers and Information System, Egyptian Chinese University, Cairo, Egypt; 5 Department of Mathematics, Faculty of Science, Islamic University of Madinah, Medina, Saudi Arabia; 6 Department of Mathematics, College of Science, King Khalid University, Abha, Saudi Arabia; 7 Department of Basic Science, Higher Institute of Computer Science and Information Systems, Fifth Settlement, Cairo, Egypt

**Keywords:** The second-type Green-Naghdi thermoelastic theory, Nonlinear thermoelasticity, Laser pulse heating, Dependent on thermal variations, Analytical solution, Thermal stress analysis, Engineering, Materials science, Mathematics and computing, Physics

## Abstract

This study presents a detailed analytical exploration of exact wave solutions under the Green-Naghdi type II (G-N II) thermoelastic theory, emphasizing the role of laser pulse interactions and temperature-sensitive material properties. By employing the Modified Extended Direct Algebraic (MEDA) method, the work derives precise closed-form solutions for the governing thermo-mechanical equations, elucidating the coupled thermal and elastic wave propagation in deformable solids. The central theme of the investigation revolves around how the presence of laser pulse phenomena and the variation of material parameters with temperature significantly influence the response of thermoelastic media. These factors are shown to be critical in dictating the behavior of stress and thermal distributions under varying mechanical and thermal loading conditions. The MEDA approach proves to be a powerful and adaptable mathematical tool, as it facilitates the construction of broad families of wave solutions, each containing arbitrary constants that allow for modeling a diverse array of physical scenarios. By incorporating these free parameters into the solution structures, the study greatly expands the set of exact solutions available, enabling a more comprehensive analysis of wave behavior under distinct environmental and loading circumstances. The derived solutions provide meaningful insights into how wave propagation is affected in thermoelastic systems, particularly in terms of variations in wave velocity, attenuation, and energy transport mechanisms when subject to laser-induced thermal excitation and non-constant material characteristics. In addition to the analytical development, the research supports its theoretical predictions through graphical depictions of various important physical quantities, including stress, displacement, and temperature profiles. A comprehensive parametric investigation is conducted to rigorously examine the influence of multiple key parameters, including laser pulse duration, material properties, and exposure time, in addition to pulse intensity. These visualizations clearly reveal the complex and dynamic interplay between thermal and mechanical influences, especially when external sources such as laser pulses are introduced. This study enhances the comprehension of thermoelastic wave propagation in modern materials exhibiting temperature-dependent behavior.

## Introduction

The Green-Naghdi Type II (G-N II) theory represents a significant advancement in modeling thermo-mechanical interactions in solids. Unlike traditional Fourier-based heat conduction models—which rely on the physically unrealistic assumption of infinite thermal wave speed–the G-N II framework introduces finite thermal propagation speeds. This critical improvement enables more accurate predictions of material responses under rapid temperature changes and dynamic mechanical loading. A key characteristic of the G-N II model is its non-dissipative heat conduction equation, which results in a hyperbolic system. This formulation supports the propagation of thermal waves without damping, maintaining their amplitude over time and space. This behavior not only improves theoretical consistency, but also matches better with experimental data, especially in high-speed thermal processes where energy dissipation is minimal. The G-N II model thus serves as a powerful tool for predicting and analyzing material responses under non-classical thermoelastic conditions, particularly where the assumption of instantaneous thermal diffusion fails. Its applications span numerous high-tech and scientific domains. In advanced materials engineering, for instance, it provides deeper insights into how materials behave under thermal shocks, high-frequency heating, or pulsed energy sources, offering valuable predictions that support the design of resilient and high-performance systems. For a complete understanding of this theory and its practical implications, the reader is referred to sources^1–12^. The study of laser pulse effects on thermoelastic materials exhibiting temperature-dependent properties has gained significant interest in applied mathematics and engineering. This is largely due to its wide-ranging applications in fields like microelectronics, biomedical engineering, and advanced materials research. Thermoelasticity describes the coupling between thermal and mechanical phenomena in solid media, and when a laser pulse is introduced, it serves as an intense and highly localized source of heat. This abrupt thermal input gives rise to a complex interplay of temperature-induced deformations and stress waves. The modeling of such phenomena becomes especially intricate when the material properties vary with temperature, as this introduces nonlinearities and non-uniformities in both space and time. Consequently, laser pulse exposure induces dynamically evolving stress and temperature fields, necessitating the use of sophisticated mathematical tools for accurate description. Researchers often utilize innovative analytical methods, such as exact wave solutions, along with advanced numerical simulations to analyze the resulting thermal gradients, stress distributions, and elastic responses. Accurately capturing these phenomena is vital for understanding and predicting material behavior under extreme or rapidly changing thermal conditions. Such insights are critical for the design and reliability of components exposed to thermal shocks or cyclic heating, as they directly impact the mechanical performance and long-term durability of these systems. For further elaboration of these topics, the readers are referred to sources^13–18,20–30^. The incorporation of temperature-dependent properties in thermoelastic models plays a pivotal role in achieving precise predictions of material response under coupled thermal-mechanical loading. This is particularly crucial for materials whose properties—including thermal conductivity, elastic moduli, specific heat, thermal expansion coefficients, and density—vary substantially with temperature. Such variations introduce nonlinearities into the thermoelastic governing equations, complicating their mathematical treatment but improving their physical fidelity^31–36^. This aspect is especially vital in applications subjected to extreme thermal transients, such as laser material processing, aerospace components, and nuclear reactor systems. In these scenarios, materials often experience rapid temperature fluctuations, leading to strongly coupled, nonlinear interactions between heat transfer and mechanical deformation. The temperature dependence of material parameters results in spatially non-uniform and temporally evolving thermal stresses, which may induce complex phenomena like stress localization, nonlinear elastic wave dynamics, and alterations in structural stability. Furthermore, temperature-dependent properties affect the propagation characteristics of thermoelastic waves, modifying their velocity and attenuation, thereby influencing energy dissipation mechanisms^31–34^. To address the analytical challenges posed by these nonlinearities, researchers employ advanced mathematical techniques, such as perturbation analysis^37^, the normal mode method^38,39^, and other sophisticated solution approaches^40^, the incorporation of temperature-dependent properties within the G-N II thermoelastic framework significantly improves the model’s accuracy in predicting material behavior. This advancement allows for more realistic modeling of thermal stresses and expands the theory’s utility to diverse materials, particularly in high-temperature environments. As a result, temperature-sensitive thermoelastic analyzes become critical to optimizing the design of thermally stable structures, ensuring their durability under extreme thermal loads. Numerous studies have validated the relevance of the G-N II theory in modeling thermoelastic wave propagation, responses in microstructured and functionally graded materials, and applications across diverse temperature-sensitive systems (see^41–46^). The pursuit of exact analytical solutions plays a vital role in the realms of applied science and mathematical physics, as these solutions offer a clear and rigorous representation of physical systems without resorting to computational approximations or numerical methods. By providing explicit expressions, exact solutions serve as essential tools for understanding the core dynamics of Partial Differential Equations (PDEs) that model various phenomena, including thermal conduction, fluid motion, plasma behavior, thermoelastic interactions, and nonlinear wave propagation^47^. Such precise solutions are particularly valuable when dealing with Nonlinear Partial Differential Equations (NLPDEs), which commonly arise in complex media and systems. In these contexts, exact solutions facilitate the analysis of critical aspects like energy distribution, wave structure, and stability characteristics, allowing researchers to investigate how different variables and parameters influence system behavior. To derive these solutions, a wide range of analytical techniques has been developed over the years. Notable among them are the Hirota bilinear method^48^, the inverse scattering transform^49,50^, and the MEDA method^51–53^, each offering a systematic framework to tackle different classes of nonlinear equations. These techniques not only yield explicit solutions but also uncover deeper mathematical structures, such as symmetry properties, conservation laws, and integrability conditions, enriching the theoretical foundation of the models under consideration. Moreover, recent advances in these methodologies have enabled the discovery of new classes of exact solutions in a variety of disciplines, including biological modeling^54^, optical communication systems like nonlinear optical fibers^55^, and quantum mechanical systems^56^. These developments underscore the broad applicability and practical importance of exact solutions across diverse scientific and technological domains. As a result, the ongoing effort to uncover exact analytical solutions continues to be a cornerstone of modern mathematical inquiry. It not only deepens theoretical insights but also provides benchmark solutions that validate numerical schemes, guide experimental design, and support the development of innovative technologies in engineering and applied physics.

The physical phenomenon investigated in this study concerns the interaction of pulsed laser heating with thermoelastic media whose properties vary with temperature. A laser pulse delivers a sudden and spatially localized energy input that produces steep thermal gradients, which in turn induce elastic deformations and stress waves through thermo-mechanical coupling. The situation becomes more intricate when the thermal and mechanical material parameters are temperature-dependent, as this introduces nonlinearities and spatial non-uniformities into the response. Understanding these coupled effects is of considerable significance because they directly influence the stability, durability, and performance of materials subjected to rapid thermal loading. Such processes are central to numerous modern applications, including microelectronics cooling and reliability, biomedical treatments involving laser–tissue interactions, aerospace structures under thermal shock, and nuclear components exposed to transient heating. By analyzing this phenomenon within the framework of the G–N II thermoelastic theory, which accounts for finite thermal wave propagation and avoids the limitations of Fourier-based diffusion, the present study provides physically consistent insights into how temperature-dependent material properties govern stress, displacement, and thermal wave behavior under laser excitation.

This research is dedicated to a thorough investigation of the pronounced impact that laser pulse phenomena exert on thermoelastic media characterized by temperature-dependent properties. The examination is conducted within the framework of the G-N II theory of thermoelasticity, with the MEDA technique serving as the primary analytical approach. A detailed exposition of the MEDA method will be provided in later sections, highlighting its theoretical foundation and its robustness in addressing NLPDEs that describe intricate physical interactions. The study undertakes a comprehensive analysis of the various forms of solutions derived via this technique, aiming to elucidate the behavior of thermoelastic systems subjected to various physical conditions. This includes a dual focus on both thermal and mechanical responses, with special attention to the interplay between laser pulse effects and the thermal sensitivity of material parameters. To complement the analytical findings, the study incorporates a series of two-dimensional graphical illustrations. These plots are instrumental in revealing underlying trends and behaviors, offering valuable visual representation of the coupled thermo-mechanical interactions, and deepening the understanding of the complex dynamics encapsulated within the model.

## Summary of the methodology

In this section, we present a concise description of the MEDA method for solving NLPDE (see^57–59^). Consider the general form:

1 $$\begin{aligned} F\left( \theta ,\theta _{\tilde{t}},\theta _{\tilde{x}},\theta _{\tilde{t}\tilde{t}},\theta _{\tilde{x}\tilde{x}},\theta _{\tilde{t}\tilde{x}},\dots \right) =0. \end{aligned}$$

Here, $\theta = \theta (\tilde{t}, \tilde{x})$ denotes the unknown function depending on temporal and spatial variables.

Step 1: traveling wave transformation

To simplify the governing NLPDE, we introduce the traveling wave transformation:

2 $$\begin{aligned} z = b_1(\tilde{t}) + b_2(\tilde{t})\,\tilde{x}, \quad \theta (\tilde{t}, \tilde{x}) = \bar{T}(z). \end{aligned}$$

This transformation reduces the PDE into an ordinary differential equation (ODE) by combining the independent variables into a single wave variable.

Step 2: Reduction to an ordinary differential equation

Applying the above transformation converts the original equation into the following nonlinear ODE:

3 $$\begin{aligned} \mathring{R}\left( \bar{T}, \frac{d\bar{T}}{dz}, \frac{d^2\bar{T}}{dz^2}, \frac{d^3\bar{T}}{dz^3}, \dots \right) =0. \end{aligned}$$

Step 3: Solution ansatz

The MEDA method assumes that the solution of the reduced ODE can be written as:

4 $$\begin{aligned} \bar{T}(z) = \sum _{i=-N}^{N} \beta _i \, \Phi (z)^i, \end{aligned}$$

where $\beta _i$ are constants to be determined, and $\Phi (z)$ satisfies the auxiliary equation:

5 $$\begin{aligned} \Phi '(z) = \sqrt{g_0 + g_1 \Phi + g_2 \Phi ^2 + g_3 \Phi ^3 + g_4 \Phi ^4 + g_6 \Phi ^6}. \end{aligned}$$

Step 4: Determination of the parameter *N*

The positive integer *N* is determined using the homogeneous balance principle by equating the highest-order derivative term with the dominant nonlinear term in the reduced ODE.

Step 5: Algebraic system construction

Substituting the assumed solution and the auxiliary equation into the reduced ODE and collecting coefficients of like powers of $\Phi (z)$ yields a system of nonlinear algebraic equations.

Step 6: Construction of exact solutions

Solving the resulting algebraic system provides explicit expressions for the unknown parameters, which can then be substituted back to construct exact analytical solutions of the original NLPDE.

## The basic equations

This section outlines the theoretical framework governing the interaction of laser pulses with thermoelastic materials, accounting for temperature-dependent properties. Based on the Green–Naghdi (G–N) theory of Type II thermoelasticity, the governing equations are derived to model the system’s nonlinear behavior. To enhance computational and analytical tractability, these equations are non-dimensionalized, revealing key dimensionless parameters that dictate the underlying thermoelastic dynamics. A critical step in the analysis involves the application of a moving wave transformation, defined by a suitable coordinate shift. This technique converts the original system of coupled partial differential equations (PDEs) into a simplified set of ordinary differential equations (ODEs). Such a reduction preserves the essential physics of wave propagation while facilitating analytical solutions. The transformation introduces a unified independent variable, $\omega = x - v t$, representing a traveling wave with velocity $v$. This approach not only streamlines the mathematical treatment but also provides a robust framework for examining the transient thermoelastic response induced by laser irradiation. The resulting ODE system enables a detailed investigation of thermal and mechanical wave interactions, offering insights into energy dissipation and stress distribution in the material. The displacement field within the system can be mathematically described using the following components:

$$\begin{aligned} u=u\left( x,t\right) ,\quad v=w=0. \end{aligned}$$

The derived analytical expression for the stress component $\tau _{xx}$ takes the following form:

6 $$\begin{aligned} \tau _{xx}=\left( 2\mu +\lambda \right) u_{,x}-\beta \theta , \end{aligned}$$

in this context, the notation $\left( , \right)$ denotes differentiation. The system’s equation of motion can be expressed in the following form^60^:

7 $$\begin{aligned} \tau _{xx,x}=\rho u_{,tt}, \end{aligned}$$

The heat conduction equation, derived from the G-N II theory, is presented in the following form^61^:

8 $$\begin{aligned} \left( k\theta _{,tx}\right) _{,x}=\frac{\partial ^{2}}{\partial t^{2}} \left( \rho c_{e}\theta +\beta T_{0}u_{,x}\right) +\frac{\partial }{\partial t}\left( \rho Q\right) , \end{aligned}$$

where $\frac{\left| \theta \right| }{T_{0}}\prec \prec 1.$ The heat energy is introduced into the system through a laser pulse, which serves as a distributed heat source across a unit volume. The specific form of this heat source is represented as follows^62^:

$$\begin{aligned} Q\left( x,t\right) =\frac{I_{0}t\gamma }{2\pi r^{2}t_{0}^{2}}e^{\left( - \frac{x^{2}}{r^{2}}-\frac{t}{t_{0}}\right) }. \end{aligned}$$

We presume that:

9 $$\begin{aligned} \left( \rho ,\lambda ,k,\beta ,\mu \right) =\left( \mathring{\rho },\mathring{\lambda },\mathring{k},\mathring{\beta },\mathring{\mu }\right) f\left( \theta \right) , \end{aligned}$$

Within this formulation, the function $f(\theta )$ exhibits continuity across the interval $[0, \infty )$. Furthermore, the parameters $\mathring{\rho }$, $\mathring{\lambda }$, $\mathring{k}$, $\mathring{\beta }$, and $\mathring{\mu }$ are treated as constants with fixed values. By inserting Eq. (9) into Eqs. (6), (7), and (8), we deduce that:

10 $$\begin{aligned} \tau _{xx}=\left( 2\mathring{\mu }+\mathring{\lambda }\right) f\left( \theta \right) u_{,x}-\mathring{\beta }f\left( \theta \right) \theta , \end{aligned}$$

11 $$\begin{aligned} \tau _{xx,x}=\mathring{\rho }f\left( \theta \right) u_{,tt}, \end{aligned}$$

12 $$\begin{aligned} \left[ \mathring{k}f\left( \theta \right) \theta _{,tx}\right] _{,x}=\frac{ \partial ^{2}}{\partial t^{2}}\left[ c_{e}\mathring{\rho }f\left( \theta \right) \theta +\mathring{\beta }f\left( \theta \right) T_{0}u_{,x}\right] + \frac{\partial }{\partial t}\left( \mathring{\rho }f\left( \theta \right) Q\right) . \end{aligned}$$

This research adopts a non-dimensionalization procedure to transform the governing equations into a simplified form. By introducing dimensionless variables, the physical quantities are normalized, leading to a more compact mathematical representation that eases analytical and numerical handling. This methodology not only diminishes the intricacy of the original equations but also promotes computational efficiency. Furthermore, it provides a universal framework, extending the relevance of the findings to diverse physical conditions. The detailed expressions of these dimensionless variables are defined as follows:

13 $$\begin{aligned} \tilde{\textit{t}}=\eta ^{2}\varpi t,\,\tilde{\tau }_{xx}=\frac{\tau _{xx}}{2 \mathring{\mu }+\mathring{\lambda }},\left( \tilde{u},\tilde{x} \right) =\eta \varpi \left( u,x\right) ,\tilde{\theta }=\frac{ \theta }{T_{0}},\tilde{Q}=\frac{Q}{\eta ^{4}\varpi }. \end{aligned}$$

where $\eta ^{2}\mathring{\rho }=2\mathring{\mu }+\mathring{\lambda }$ and $\mathring{\rho }c_{e}=\varpi \mathring{k}.$ We clear that The dimensionless time $\tilde{t}$ is scaled using a characteristic thermoelastic time parameter, while the stress tensor $\tilde{\tau }_{xx}$ is normalized by the effective elastic modulus $(2 \mathring{\mu }+\mathring{\lambda })$. The displacement and spatial coordinate $( \tilde{u},\tilde{x} )$ are scaled by a characteristic wave-related parameter, reflecting the propagation of thermoelastic disturbances. The temperature $\tilde{\theta }$ is defined relative to the reference temperature $T_{0}$, representing the normalized temperature variation and the heat source $\tilde{Q}$ is scaled to indicate its relative intensity within the medium. By combining Eq. (13) with Eq. (11) and utilizing the relation from Eq. (10), the resulting expression becomes:

14 $$\begin{aligned} f\left( \tilde{\theta }\right) \left[ \tilde{u}_{,\tilde{x}\tilde{x}}-\tilde{u }_{,\tilde{\textit{t}}\tilde{\textit{t}}}-l_{1}\tilde{\theta }_{,\tilde{x}}\right] +f^{^{\prime }}\left( \tilde{\theta }\right) \left[ \tilde{u}_{,\tilde{x}}\tilde{\theta }_{,\tilde{x}}-l_{1}\tilde{\theta }\tilde{\theta }_{,\tilde{x}}\right] =0, \end{aligned}$$

where $$f^{^{\prime }}\left( \tilde{\theta }\right) =\frac{df\left( \tilde{ \theta }\right) }{d\tilde{\theta }}$$ and $$l_{1}=\frac{T_{0}\mathring{\beta }}{2 \mathring{\mu }+\mathring{\lambda }}.$$

By substituting Eq. (13) into Eq. (12), we obtain:

15 $$\begin{aligned} f\left( \tilde{\theta }\right) \left[ \tilde{\theta }_{,\tilde{x}\tilde{x} \tilde{\textit{t}}}-\tilde{\theta }_{,\tilde{\textit{t}}\tilde{\textit{t}}}-l_{2}\tilde{u}_{,\tilde{x} \tilde{\textit{t}}\tilde{\textit{t}}}-l_{3}\right] +f^{^{\prime }}\left( \tilde{\theta }\right) \left[ \tilde{\theta }_{,\tilde{x}\tilde{\textit{t}}}\tilde{\theta }_{,\tilde{x}}- \tilde{\theta }_{,\tilde{\textit{t}}\tilde{\textit{t}}}\tilde{\theta }-l_{2}\tilde{\theta }_{,\tilde{\textit{t}}\tilde{\textit{t}}}\tilde{u}_{,\tilde{x}}-l_{4}\right] +f^{^{\prime \prime }}\left( \tilde{\theta }\right) \left[ \left( \tilde{\theta }_{,\tilde{\textit{t}} }\right) ^{2}\tilde{\theta }-l_{2}\left( \tilde{\theta }_{,\tilde{\textit{t}}}\right) ^{2}\tilde{u}_{,\tilde{x}}\right] =0, \end{aligned}$$

where $$l_{2}=\frac{\mathring{\beta }}{\varpi \mathring{k}},$$ $$l_{3}=\frac{ \mathring{\rho }\eta ^{2}}{\mathring{k}T_{0}\varpi }\dot{Q},$$ $$l_{4}=\frac{ \mathring{\rho }\eta ^{2}}{\mathring{k}T_{0}\varpi }Q,$$ and $$f^{^{\prime \prime }}\left( \tilde{\theta }\right) =\frac{d^{2}f\left( \tilde{\theta } \right) }{d\tilde{\theta }^{2}}.$$

By inserting Eq. (13) into Eq. (10), the resulting expression becomes:

16 $$\begin{aligned} \tilde{\tau }_{xx}=f\left( \tilde{\theta }\right) \left[ \tilde{u}_{,\tilde{x} }-l_{1}\tilde{\theta }\right] . \end{aligned}$$

In line with established thermoelasticity models, the governing function employed in this study is defined as follows^63^:

$$\begin{aligned} f\left( \theta \right) =1-\frac{h }{T_{0}}\theta , \end{aligned}$$

The symbol *h* denotes a fixed constant in this context. By introducing the dimensionless variables into the aforementioned equation, we derive the following expression:

17 $$\begin{aligned} f\left( \tilde{\theta }\right) =1-h \tilde{\theta }. \end{aligned}$$

By substituting Eq. (17) into Eqs. (14), (15), and (16), we obtain:

18 $$\begin{aligned} \tilde{u}_{,\tilde{x}\tilde{x}}-\tilde{u}_{,\tilde{\textit{t}}\tilde{\textit{t}}}-l_{1}\tilde{ \theta }_{,\tilde{x}}-h \tilde{\theta }\tilde{u}_{,\tilde{x}\tilde{x}}+h \tilde{\theta }\tilde{u}_{,\tilde{\textit{t}}\tilde{\textit{t}}}+2h l_{1}\tilde{\theta }\tilde{ \theta }_{,\tilde{x}}-h \tilde{u}_{,\tilde{x}}\tilde{\theta }_{,\tilde{x}}=0, \end{aligned}$$

19 $$\begin{aligned} \tilde{\theta }_{,\tilde{x}\tilde{x}\tilde{\textit{t}}}-\tilde{\theta }_{,\tilde{\textit{t}} \tilde{\textit{t}}}-l_{2}\tilde{u}_{,\tilde{x}\tilde{\textit{t}}\tilde{\textit{t}}}-h \tilde{\theta } \tilde{\theta }_{,\tilde{x}\tilde{x}\tilde{\textit{t}}}+2h \tilde{\theta }\tilde{ \theta }_{,\tilde{\textit{t}}\tilde{\textit{t}}}+h l_{2}\tilde{\theta }\tilde{u}_{,\tilde{x} \tilde{\textit{t}}\tilde{\textit{t}}}-h \tilde{\theta }_{,\tilde{x}\tilde{\textit{t}}}\tilde{\theta }_{,\tilde{x}}+h l_{2}\tilde{\theta }_{,\tilde{\textit{t}}\tilde{\textit{t}}}\tilde{u}_{,\tilde{x} }+h l_{3}\tilde{\theta }+h l_{4}-l_{3}=0, \end{aligned}$$

20 $$\begin{aligned} \mathring{\tau }_{zz}=\tilde{u}_{,\tilde{x}}-l_{1}\tilde{\theta }-h \tilde{ \theta }\tilde{u}_{,\tilde{x}}+h l_{1}\left( \tilde{\theta }\right) ^{2}. \end{aligned}$$

We utilize the subsequent format for the traveling wave transformation:

21 $$\begin{aligned} \tilde{u}\left( \tilde{x},\tilde{\textit{t}}\right) =U\left( \omega \right) ,\quad \theta \left( \tilde{x},\tilde{\textit{t}}\right) =\theta \left( \omega \right) , \quad \omega =b_{1}\left( \tilde{\textit{t}} \right) +b_{2}\left( \tilde{\textit{t}}\right) \tilde{x}. \end{aligned}$$

By applying Eq. (21) to Eqs. (18), (19), and (20), we obtain:

22 $$\begin{aligned} a_{1}U^{^{\prime \prime }}-a_{2}U^{^{\prime }}-a_{3}\theta ^{^{\prime }}-a_{4}\theta U^{^{\prime \prime }}+a_{5}\theta U^{^{\prime }}+a_{6}\theta \theta ^{^{\prime }}-a_{7}U^{^{\prime }}\theta ^{^{\prime }}=0, \end{aligned}$$

23 $$\begin{aligned} 0= & a_{8}\theta ^{^{\prime \prime \prime }}+a_{9}\theta ^{^{\prime \prime }}-a_{10}\theta ^{^{\prime }}-a_{11}U^{^{\prime \prime \prime }}-a_{12}U^{^{\prime \prime }}-a_{13}U^{^{\prime }}+a_{14}\theta \theta ^{^{\prime \prime \prime }}+a_{15}\theta \theta ^{^{\prime \prime }}+a_{16}\theta \theta ^{^{\prime }}+a_{17}\theta U^{^{\prime \prime \prime }} \nonumber \\ & +a_{18}\theta U^{^{\prime \prime }}+a_{19}\theta U^{^{\prime }}+a_{17}U^{^{\prime }}\theta ^{^{\prime \prime }}-a_{14}\theta ^{^{\prime }}\theta ^{^{\prime \prime }}+a_{20}U^{^{\prime }}\theta ^{^{\prime }}-a_{21}\left( \theta ^{^{\prime }}\right) ^{2}+a_{22}\theta +a_{23}, \end{aligned}$$

24 $$\begin{aligned} \tilde{\tau }_{xx}=b_{2}U^{^{\prime }}-a_{24}\theta U^{^{\prime }}-l_{1}\theta +a_{25}\left( \theta \right) ^{2}, \end{aligned}$$

where $$\theta ^{^{\prime }}=\frac{d\theta }{d\omega }$$, $$\dot{b}_{1}=\frac{ db_{1}}{d\tilde{\textit{t}}},$$ $$a_{1}=b_{2}^{2}-\left( \dot{b}_{1}+\dot{b}_{2}\tilde{x }\right) ^{2},$$ $$a_{2}=\ddot{b}_{1}+\ddot{b}_{2}\tilde{x},$$ $$a_{3}=l_{1}b_{2},$$ $$a_{4}=h a_{1},$$

$$a_{5}=h a_{2},$$ $$a_{6}=2h l_{1}b_{2},$$ $$a_{7}=h b_{2}^{2},$$ $$a_{8}=b_{2}^{2}\left( \dot{b}_{1}+\dot{b}_{2}\tilde{x}\right) ,$$ $$a_{9}=2b_{2}\dot{b}_{2}-\left( \dot{b}_{1}+\dot{b}_{2}\tilde{x}\right) ^{2},$$

$$a_{10}=\ddot{b}_{1}+\ddot{b}_{2}\tilde{x},$$ $$a_{11}=l_{2}b_{2}\left( \dot{b} _{1}+\dot{b}_{2}\tilde{x}\right) ^{2},$$ $$a_{12}=2l_{2}\dot{b}_{2}\left( \dot{ b}_{1}+\dot{b}_{2}\tilde{x}\right) +l_{2}b_{2}\left( \ddot{b}_{1}+\ddot{b} _{2}\tilde{x}\right) ,$$ $$a_{13}=l_{2}\ddot{b}_{2},$$

$$a_{14}=h a_{8},$$ $$a_{15}=2h \left( \dot{b}_{1}+\dot{b}_{2}\tilde{x} \right) ^{2}-2h b_{2}\dot{b}_{2},$$ $$a_{16}=2h a_{10},$$ $$a_{17}=h a_{11},$$ $$a_{18}=h a_{12},$$ $$a_{19}=h a_{13},$$

$$a_{20}=h l_{2}b_{2}\left( \ddot{b}_{1}+\ddot{b}_{2}\tilde{x}\right) ,$$ $$a_{21}=h b_{2}\dot{b}_{2},$$ $$a_{22}=h l_{3},$$ $$a_{23}=h l_{4}-l_{3},$$ $$a_{24}=h b_{2},$$ $$a_{25}=h l_{1}.$$

## Retrieve explicit analytical solutions for the given model

Suppose that:

25 $$\begin{aligned} U=\zeta \ \theta , \end{aligned}$$

thus, Eqs. (22) and (23) can be restructured and expressed in the following form:

26 $$\begin{aligned} \zeta a_{1}\theta ^{^{\prime \prime }}-\left( \zeta a_{2}+a_{3}\right) \theta ^{^{\prime }}-\zeta a_{4}\theta \theta ^{^{\prime \prime }}+\left( \zeta a_{5}+a_{6}\right) \theta \theta ^{^{\prime }}-\zeta a_{7}\left( \theta ^{^{\prime }}\right) ^{2}=0, \end{aligned}$$

27 $$\begin{aligned} 0= & \left[ a_{8}-\zeta a_{11}\right] \theta ^{^{\prime \prime \prime }}+ \left[ a_{9}-\zeta a_{12}\right] \theta ^{^{\prime \prime }}-\left[ a_{10}+\zeta a_{13}\right] \theta ^{^{\prime }}+\left[ a_{14}+\zeta a_{17} \right] \theta \theta ^{^{\prime \prime \prime }}+\left[ a_{15}+\zeta a_{18} \right] \theta \theta ^{^{\prime \prime }} \nonumber \\ & +\left[ a_{16}+\zeta a_{19}\right] \theta \theta ^{^{\prime }}+\left[ \zeta a_{17}-a_{14}\right] \theta ^{^{\prime }}\theta ^{^{\prime \prime }}+ \left[ \zeta a_{20}-a_{21}\right] \left( \theta ^{^{\prime }}\right) ^{2}+a_{22}\theta +a_{23}. \end{aligned}$$

Using Eq. (26) in Eq. (27), one realizes

28 $$\begin{aligned} a_{27}\theta ^{^{\prime \prime \prime }}+a_{28}\theta ^{^{\prime \prime }}-a_{29}\theta ^{^{\prime }}+a_{30}\theta \theta ^{^{\prime \prime \prime }}+a_{31}\theta \theta ^{^{\prime \prime }}+a_{32}\theta \theta ^{^{\prime }}+a_{33}\theta ^{^{\prime }}\theta ^{^{\prime \prime }}+a_{22}\theta +a_{23}=0, \end{aligned}$$

where $$a_{26}=\frac{\left[ \zeta a_{20}-a_{21}\right] }{\zeta a_{7}},$$ $$a_{27}=a_{8}-\zeta a_{11},$$ $$a_{28}=a_{9}-\zeta a_{12}+\zeta a_{1}a_{26},$$ $$a_{29}=a_{10}+\zeta a_{13}-\left( \zeta a_{2}+a_{3}\right) a_{26},$$ $$a_{30}=a_{14}+\zeta a_{17},$$ $$a_{31}=a_{15}+\zeta a_{18}-\zeta a_{4}a_{26},$$$$a_{32}=a_{16}+\zeta a_{19}+\left( \zeta a_{5}+a_{6}\right) a_{26},$$ $$a_{33}=\zeta a_{17}-a_{14}.$$

The general solution is rigorously formulated by methodically applying the balancing principle between the dominant dispersive term and the highest-order nonlinear term in Eq.(28). The following complete statement for the solution structure is obtained using this basic approach:

29 $$\begin{aligned} \theta (z )=\beta _0+\beta _1 \Phi (z )+\beta _2 \Phi ^2 (z )+\frac{\beta _{-1}}{\Phi (z )}+\frac{\beta _{-2}}{\Phi ^2 (z )}, \end{aligned}$$

The coefficients $\beta _{-2}, \beta _{-1}, \beta _{0}, \beta _{1},$ and $\beta _{2}$ are real-valued constants to be determined. They must satisfy the non-degeneracy condition $\beta _{2}^{2} + \beta _{-2}^{2} \ne 0$, which guarantees that $\beta _{2}$ and $\beta _{-2}$ do not vanish simultaneously. By substituting Eq. (29) and Eq. (5) into Eq. (28), we collect like terms in powers of $\Phi (z)^{i}$ (for $i = 0, 1, 2, \dots$) and equate each coefficient to zero. This yields a system of algebraic equations, which can be solved computationally using symbolic computation software such as Maple or Mathematica. The resulting solutions are obtained as follows: Set(1): If $g_{0}= g_{1}= g_{3}=g_{6}=0$, the following results are obtained:

$$(1.1)\hspace{0.3cm}\beta _{-1}=\beta _1=\beta _2=0,\hspace{0.3cm}\beta _{-2}=\frac{a_{23} a_{31}-a_{27} \left( a_{27}-2 a_{28}\right) g_2}{2 \left( 2 a_{27}-a_{28}\right) a_{31} g_4},\hspace{0.3cm}\beta _0=-\frac{a_{27}}{2 a_{31}},\hspace{0.3cm}a_{33}=-\frac{6 a_{31} g_2+a_{32}}{4 g_2},$$

$$a_{29}=\frac{3 a_{27} g_2 \left( -20 a_{28} a_{31} g_2-a_{27} \left( a_{32}-10 a_{31} g_2\right) \right) -a_{23} a_{31} \left( 30 a_{31} g_2+a_{32}\right) }{4 \left( 2 a_{27}-a_{28}\right) a_{31} g_2},\hspace{0.3cm}a_{33}=-\frac{6 a_{31} g_2+a_{32}}{4 g_2},\hspace{0.3cm}a_{30}=2 a_{31},$$

$$a_{22}=\frac{\left( a_{27}-2 a_{28}\right) {}^2 g_2+3 a_{23} a_{31}}{2 a_{27}-a_{28}}.$$

An analysis of the system (1.1) yields distinct classes of solutions, among which hyperbolic solutions emerge under the conditions $g_{2}>0$ , $a_{31} \ne 0$ and $\left( 2 a_{27}-a_{28}\right) g_2\ne 0$. The existence and behavior of these solutions are thus governed by the interplay of these critical parameters, as systematically explored in the subsequent analysis of the system:

30 $$\begin{aligned} & \tilde{\theta }_{1.1}=\frac{1}{2 a_{31}}\left( \frac{\mathcal {H} \cosh ^2 \left( \sqrt{g_2} (b_{1}\left( \tilde{\textit{t}} \right) +b_{2}\left( \tilde{\textit{t}}\right) \tilde{x})\right) }{\left( 2 a_{27}-a_{28}\right) g_2}-a_{27}\right) , \end{aligned}$$

31 $$\begin{aligned} & \tilde{u}_{1.1}=\frac{\xi }{2 a_{31}}\left( \frac{\mathcal {H} \cosh ^2 \left( \sqrt{g_2} (b_{1}\left( \tilde{\textit{t}} \right) +b_{2}\left( \tilde{\textit{t}}\right) \tilde{x})\right) }{\left( 2 a_{27}-a_{28}\right) g_2}-a_{27}\right) , \end{aligned}$$

according to the previous relations, the stress tensor is formulated as:

32 $$\begin{aligned} & \tilde{\tau }_{xx}=(\frac{b_2 \xi \mathcal {H} \sinh \left( \sqrt{g_2} (b_{1}\left( \tilde{\textit{t}} \right) +b_{2}\left( \tilde{\textit{t}}\right) \tilde{x})\right) \cosh \left( \sqrt{g_2} (b_{1}\left( \tilde{\textit{t}} \right) +b_{2}\left( \tilde{\textit{t}}\right) \tilde{x})\right) }{\left( 2 a_{27}-a_{28}\right) a_{31} \sqrt{g_2}}-\frac{l_1 }{2 a_{31}}(\frac{\mathcal {H} \cosh ^2\left( \sqrt{g_2} (b_{1}\left( \tilde{\textit{t}} \right) +b_{2}\left( \tilde{\textit{t}}\right) \tilde{x})\right) }{\left( 2 a_{27}-a_{28}\right) g_2}\nonumber \\ & -a_{27})- \frac{l_1 }{2 a_{31}} \left( \frac{\mathcal {H} \cosh ^2\left( \sqrt{g_2} (b_{1}\left( \tilde{\textit{t}} \right) +b_{2}\left( \tilde{\textit{t}}\right) \tilde{x})\right) }{\left( 2 a_{27}-a_{28}\right) g_2}-a_{27}\right) +\frac{a_{25}^2}{4 a_{31}^2} \left( \frac{\mathcal {H} \cosh ^2\left( \sqrt{g_2} (b_{1}( \tilde{\textit{t}}) +b_{2}\left( \tilde{\textit{t}}\right) \tilde{x})\right) }{\left( 2 a_{27}-a_{28}\right) g_2}-a_{27}\right) \nonumber \\ & -\frac{a_{24} \xi \mathcal {H} \sinh \left( \sqrt{g_2} (b_{1}\left( \tilde{\textit{t}} \right) +b_{2}\left( \tilde{\textit{t}}\right) \tilde{x})\right) \cosh \left( \sqrt{g_2} (b_{1}\left( \tilde{\textit{t}} \right) +b_{2}\left( \tilde{\textit{t}}\right) \tilde{x})\right) \left( \frac{\mathcal {H} \cosh ^2\left( \sqrt{g_2} (b_{1}\left( \tilde{\textit{t}} \right) +b_{2}\left( \tilde{\textit{t}}\right) \tilde{x})\right) }{\left( 2 a_{27}-a_{28}\right) g_2}-a_{27}\right) }{2 \left( 2 a_{27}-a_{28}\right) a_{31}^2 \sqrt{g_2}}\nonumber \\ & +\frac{a_{25} \left( \frac{\mathcal {H}\cosh ^2\left( \sqrt{g_2} (b_{1}\left( \tilde{\textit{t}} \right) +b_{2}\left( \tilde{\textit{t}}\right) \tilde{x})\right) }{\left( 2 a_{27}-a_{28}\right) g_2}-a_{27}\right) {}^2}{4 a_{31}^2}-\frac{b_2 \xi \mathcal {H} \sinh \left( \sqrt{g_2} (b_{1}\left( \tilde{\textit{t}} \right) +b_{2}\left( \tilde{\textit{t}}\right) \tilde{x})\right) \cosh \left( \sqrt{g_2} (b_{1}\left( \tilde{\textit{t}} \right) +b_{2}\left( \tilde{\textit{t}}\right) \tilde{x})\right) }{\left( 2 a_{27}-a_{28}\right) a_{31} \sqrt{g_2}}\nonumber \\ & -\frac{a_{24} \xi \mathcal {H} \sinh \left( \sqrt{g_2} (b_{1}\left( \tilde{\textit{t}} \right) +b_{2}\left( \tilde{\textit{t}}\right) \tilde{x})\right) \cosh \left( \sqrt{g_2} (b_{1}\left( \tilde{\textit{t}} \right) +b_{2}\left( \tilde{\textit{t}}\right) \tilde{x})\right) \left( \frac{\mathcal {H} \cosh ^2\left( \sqrt{g_2} (b_{1}\left( \tilde{\textit{t}} \right) +b_{2}\left( \tilde{\textit{t}}\right) \tilde{x})\right) }{\left( 2 a_{27}-a_{28}\right) g_2}-a_{27}\right) }{2 \left( 2 a_{27}-a_{28}\right) a_{31}^2 \sqrt{g_2}}, \end{aligned}$$

where $$\mathcal {H}=a_{27} \left( a_{27}-2 a_{28}\right) g_2-a_{23} a_{31}$$. $$(1.2) \hspace{0.3cm} \beta _{-1}=\beta _1=\beta _{-2}=0,\hspace{0.3cm}\beta _2=-\frac{9 a_{31} g_4}{a_{22}},\hspace{0.3cm}\beta _0=-\frac{a_{32}}{a_{22}},\hspace{0.3cm}a_{30}=-a_{33}.$$

The analysis of set (1.2) demonstrates the existence of diverse solution types. Notably, bright soliton solutions occur under the conditions $g_{2} > 0$ and $a_{22} \ne 0$. Consequently, the characteristic behavior of these solutions is fundamentally governed by the parameter values and their mutual relationships, as will be comprehensively discussed for the considered system:

33 $$\begin{aligned} & \tilde{\theta }_{1.2}=\frac{9 a_{31} g_2 \hspace{0.1cm} \text {sech}^2 \left( \sqrt{g_2} (b_{1}\left( \tilde{\textit{t}} \right) +b_{2}\left( \tilde{\textit{t}}\right) \tilde{x}\right) -a_{32}}{a_{22}}, \end{aligned}$$

34 $$\begin{aligned} & \tilde{u}_{1.2}=\frac{\xi \left( 9 a_{31} g_2\hspace{0.1cm} \text {sech}^2\left( \sqrt{g_2} (b_{1}\left( \tilde{\textit{t}} \right) +b_{2}\left( \tilde{\textit{t}}\right) \tilde{x}\right) -a_{32}\right) }{a_{22}}, \end{aligned}$$

according to the previous relations, the stress tensor is formulated as:

35 $$\begin{aligned} & \tilde{\tau }_{xx}=\frac{1}{a_{22}^2} (a_{22} \left( a_{32} l_1-9 a_{31} g_2 \text {sech}^2\left( \sqrt{g_2} (b_{1}\left( \tilde{\textit{t}} \right) +b_{2}\left( \tilde{\textit{t}}\right) \tilde{x}\right) \left( 2 b_2 \sqrt{g_2} \xi \tanh \left( \sqrt{g_2} (b_{1}\left( \tilde{\textit{t}} \right) +b_{2}\left( \tilde{\textit{t}}\right) \tilde{x}\right) +l_1\right) \right) \nonumber \\ & +18 a_{24} a_{31} g_2^{3/2} \xi \tanh \left( \sqrt{g_2} (b_{1}\left( \tilde{\textit{t}} \right) +b_{2}\left( \tilde{\textit{t}}\right) \tilde{x}\right) \text {sech}^2\left( \sqrt{g_2} (b_{1}\left( \tilde{\textit{t}} \right) +b_{2}\left( \tilde{\textit{t}}\right) \tilde{x}\right) \left( 9 a_{31} g_2 \text {sech}^2\left( \sqrt{g_2} (b_{1}\left( \tilde{\textit{t}} \right) +b_{2}\left( \tilde{\textit{t}}\right) \tilde{x}\right) -a_{32}\right) \nonumber \\ & +a_{25} \left( a_{32}-9 a_{31} g_2 {{\,\textrm{sech}\,}}^2\left( \sqrt{g_2} (b_{1}\left( \tilde{\textit{t}} \right) +b_{2}\left( \tilde{\textit{t}}\right) \tilde{x}\right) \right) {}^2). \end{aligned}$$

Set(2): If $g_{0}= g_{3}= g_{4}=g_{6}=0$, the following results are obtained:

$$(2.1)\hspace{0.1cm} \beta _{-1}=\beta _{-2}=0,\hspace{0.3cm}\beta _2=\frac{a_1 g_2}{g_1},\hspace{0.3cm}\beta _0=\frac{a_1 a_{27} g_1+a_1 a_{28} g_1+2 a_{23}}{4 \left( a_{27}+2 a_{28}\right) g_2},\hspace{0.3cm}a_{33}=\frac{6 a_{31} g_2-a_{32}}{4 g_2},\hspace{0.3cm}a_{30}=-2 a_{31},$$

$$a_{29}=\frac{24 a_{27}^2 g_2^2+48 a_{27} a_{28} g_2^2-24 a_{23} a_{31} g_2-6 a_1 a_{27} a_{31} g_1 g_2+a_1 a_{27} a_{32} g_1+4 a_{23} a_{32}}{8 \left( a_{27}+2 a_{28}\right) g_2},\hspace{0.3cm}a_{22}=\frac{1}{2} \left( a_1 a_{31} g_1-4 a_{27} g_2-8 a_{28} g_2\right) .$$

An examination of the system in set (2.1) indicates the existence of multiple solution types. Specifically, hyperbolic solutions arise under the conditions $g_{2} > 0$ and $a_{27} + 2a_{28} \ne 0$. Consequently, the characteristics of these solutions are predominantly influenced by the parameter values and their interdependencies, as discussed in the subsequent analysis of the system:

36 $$\begin{aligned} & \tilde{\theta }_{2.1}=\frac{\beta _1 g_1 \left( a_{27} \sinh ^2\left( 2 \sqrt{g_2} (b_{1}\left( \tilde{\textit{t}} \right) +b_{2}\left( \tilde{\textit{t}}\right) \tilde{x})\right) +a_{28} \left( \cosh \left( 4 \sqrt{g_2} (b_{1}\left( \tilde{\textit{t}} \right) +b_{2}\left( \tilde{\textit{t}}\right) \tilde{x})\right) -2\right) \right) +2 a_{23}}{4 \left( a_{27}+2 a_{28}\right) g_2}, \end{aligned}$$

37 $$\begin{aligned} & \tilde{u}_{2.1}=\frac{\xi \left( \beta _1 g_1 \left( a_{27} \sinh ^2\left( 2 \sqrt{g_2} (b_{1}\left( \tilde{\textit{t}} \right) +b_{2}\left( \tilde{\textit{t}}\right) \tilde{x})\right) +a_{28} \left( \cosh \left( 4 \sqrt{g_2} (b_{1}\left( \tilde{\textit{t}} \right) +b_{2}\left( \tilde{\textit{t}}\right) \tilde{x})\right) -2\right) \right) +2 a_{23}\right) }{4 \left( a_{27}+2 a_{28}\right) g_2}, \end{aligned}$$

according to the previous relations, the stress tensor is formulated as:

38 $$\begin{aligned} & \tilde{\tau }_{xx}=(16 \left( a_{27}+2 a_{28}\right) {}^2 g_2^2)(8 \beta _1 \left( a_{27}+2 a_{28}\right) {}^2 b_2 g_1 g_2^{3/2} \xi \sinh \left( 4 \sqrt{g_2} (b_{1}\left( \tilde{\textit{t}} \right) +b_{2}\left( \tilde{\textit{t}}\right) \tilde{x})\right) \nonumber \\ & -4 \left( a_{27}+2 a_{28}\right) g_2 l_1 \left( \beta _1 g_1 \left( a_{27} \sinh ^2\left( 2 \sqrt{g_2} (b_{1}\left( \tilde{\textit{t}} \right) +b_{2}\left( \tilde{\textit{t}}\right) \tilde{x})\right) +a_{28} \left( \cosh \left( 4 \sqrt{g_2} (b_{1}\left( \tilde{\textit{t}} \right) +b_{2}\left( \tilde{\textit{t}}\right) \tilde{x})\right) -2\right) \right) +2 a_{23}\right) \nonumber \\ & -\beta _1 a_{24} \left( a_{27}+2 a_{28}\right) g_1 \sqrt{g_2} \xi \sinh \left( 4 \sqrt{g_2} (b_{1}\left( \tilde{\textit{t}} \right) +b_{2}\left( \tilde{\textit{t}}\right) \tilde{x})\right) (2 \beta _1 g_1 (a_{27} \sinh ^2\left( 2 \sqrt{g_2} (b_{1}\left( \tilde{\textit{t}} \right) +b_{2}\left( \tilde{\textit{t}}\right) \tilde{x})\right) \nonumber \\ & +a_{28} (cosh (4 \sqrt{g_2} (b_{1}( \tilde{\textit{t}} ) +b_{2}( \tilde{\textit{t}}) \tilde{x}))-2))+4 a_{23})+a_{25} (\beta _1 g_1 (a_{27} \sinh ^2(2 \sqrt{g_2} (b_{1}( \tilde{\textit{t}} ) +b_{2} \tilde{\textit{t}}) \tilde{x}))\nonumber \\ & +a_{28} (\cosh (4 \sqrt{g_2} (b_{1}( \tilde{\textit{t}} ) +b_{2}( \tilde{\textit{t}}) \tilde{x}))-2))+2 a_{23})^2). \end{aligned}$$

$$(2.2)\hspace{0.3cm}\beta _{-2}=\beta _{-1}=\beta _0=0,\hspace{0.3cm}\beta _1=-\frac{2 a_{23}}{\left( a_{27}+a_{28}\right) g_1},\hspace{0.3cm}a_2=-\frac{2 a_{23} g_2}{\left( a_{27}+a_{28}\right) g_1^2},\hspace{0.3cm} a_{33}=\frac{6 a_{31} g_2-a_{32}}{4 g_2},$$

$a_{30}=-2 a_{31},\hspace{0.3cm}a_{22}=\frac{-2 a_{27}^2 g_2-6 a_{28} a_{27} g_2-4 a_{28}^2 g_2-a_{23} a_{31}}{a_{27}+a_{28}},\hspace{0.3cm}a_{29}=\frac{12 a_{27}^2 g_2^2+12 a_{27} a_{28} g_2^2-6 a_{23} a_{31} g_2+a_{23} a_{32}}{4 \left( a_{27}+a_{28}\right) g_2}.$ An examination of the system in set (2.2) indicates the existence of multiple solution types. Specifically, hyperbolic solutions arise under the conditions $g_{2} > 0$ and $a_{27} + a_{28} \ne 0$. Consequently, the characteristics of these solutions are predominantly influenced by the parameter values and their interdependencies, as discussed in the subsequent analysis of the system:

39 $$\begin{aligned} & \tilde{\theta }_{2.2}=-\frac{a_{23} \left( \cosh \left( 4 \sqrt{g_2} (b_{1}( \tilde{\textit{t}} ) +b_{2}( \tilde{\textit{t}}) \tilde{x})\right) -3\right) }{4 \left( a_{27}+a_{28}\right) g_2}, \end{aligned}$$

40 $$\begin{aligned} & \tilde{u}_{2.2}=-\frac{a_{23} \xi \left( \cosh \left( 4 \sqrt{g_2} (b_{1}( \tilde{\textit{t}} ) +b_{2}( \tilde{\textit{t}}) \tilde{x})\right) -3\right) }{4 \left( a_{27}+a_{28}\right) g_2}, \end{aligned}$$

according to the previous relations, the stress tensor is formulated as:

41 $$\begin{aligned} & \tilde{\tau }_{xx}=\frac{1}{16 (a_{27}+a_{28}){}^2 g_2^2}(a_{23} (a_{23} (\cosh (4 \sqrt{g_2} (b_{1}( \tilde{\textit{t}} ) +b_{2}( \tilde{\textit{t}}) \tilde{x}))-3) (a_{25} (\cosh (4 \sqrt{g_2} (b_{1}( \tilde{\textit{t}} ) +b_{2}( \tilde{\textit{t}}) \tilde{x}))-3)\nonumber \\ & -4 a_{24} \sqrt{g_2} \xi \sinh (4 \sqrt{g_2} (b_{1}( \tilde{\textit{t}} ) +b_{2}( \tilde{\textit{t}}) \tilde{x})))-4 (a_{27}+a_{28}) g_2 (4 b_2 \sqrt{g_2} \xi \sinh (4 \sqrt{g_2} (b_{1}( \tilde{\textit{t}} ) +b_{2}( \tilde{\textit{t}}) \tilde{x}))\nonumber \\ & -l_1 (\cosh (4 \sqrt{g_2} (b_{1}( \tilde{\textit{t}} ) +b_{2}( \tilde{\textit{t}}) \tilde{x}))-3)))).\nonumber \\ \end{aligned}$$

Set(3): If $g_{3}= g_{4}=g_{6}=0,\hspace{0.3cm}g_{0}=\frac{g_1^2}{4 g_2}$, the following results are obtained: $(3.1)\hspace{0.1cm}\beta _0=\beta _{-1}=\beta _{-2}=0,\hspace{0.3cm}\beta _1=-\frac{2 a_{23}}{\left( a_{27}+2 a_{28}\right) g_1},\hspace{0.3cm}\beta _2=-\frac{2 a_{23} g_2}{\left( a_{27}+2 a_{28}\right) g_1^2},\hspace{0.3cm}a_{33}=\frac{6 a_{31} g_2-a_{32}}{4 g_2},\hspace{0.3cm}a_{22}=-2 \left( a_{27} g_2+2 a_{28} g_2\right) ,$

$a_{29}=\frac{6 a_{27}^2 g_2^2+12 a_{27} a_{28} g_2^2-6 a_{23} a_{31} g_2+a_{23} a_{32}}{2 \left( a_{27}+2 a_{28}\right) g_2},\hspace{0.3cm}a_{30}=-2 a_{31}.$ An examination of the system in set (3.1) indicates the existence of multiple solution types. Specifically, exponential solutions arise under the conditions $g_2>0$ and $g_1^2(a_{27}+2 a_{28})\ne 0$. Consequently, the characteristics of these solutions are predominantly influenced by the parameter values and their interdependencies, as discussed in the subsequent analysis of the system:

42 $$\begin{aligned} & \tilde{\theta }_{3.1}=\frac{a_{23} \left( g_1^2-4 g_2^2 e^{2 \sqrt{g_2} (b_{1}( \tilde{\textit{t}} ) +b_{2}( \tilde{\textit{t}}) \tilde{x})}\right) }{2 \left( a_{27}+2 a_{28}\right) g_1^2 g_2}, \end{aligned}$$

43 $$\begin{aligned} & \tilde{u}_{3.1}=\frac{a_{23} \xi \left( g_1^2-4 g_2^2 e^{2 \sqrt{g_2} (b_{1}( \tilde{\textit{t}} ) +b_{2}( \tilde{\textit{t}}) \tilde{x})}\right) }{2 \left( a_{27}+2 a_{28}\right) g_1^2 g_2}, \end{aligned}$$

according to the previous relations, the stress tensor is formulated as:

44 $$\begin{aligned} & \tilde{\tau }_{xx}= \frac{1}{4 \left( a_{27}+2 a_{28}\right) {}^2 g_1^4 g_2^2} (a_{23} (a_{23} (g_1^2-4 g_2^2 e^{2 \sqrt{g_2} (b_{1}( \tilde{\textit{t}} ) +b_{2}( \tilde{\textit{t}}) \tilde{x})}) (8 a_{24} g_2^{5/2} \xi e^{2 \sqrt{g_2} (b_{1}( \tilde{\textit{t}} ) +b_{2}( \tilde{\textit{t}}) \tilde{x})}\nonumber \\ & +a_{25} (g_1^2-4 g_2^2 e^{2 \sqrt{g_2} (b_{1}( \tilde{\textit{t}} ) +b_{2}( \tilde{\textit{t}}) \tilde{x})})) -2 (a_{27}+2 a_{28}) g_1^2 g_2(8 b_2 g_2^{5/2} \xi e^{2 \sqrt{g_2} (b_{1}( \tilde{\textit{t}} ) +b_{2}( \tilde{\textit{t}}) \tilde{x})}+l_1 (g_1^2\nonumber \\ & -4 g_2^2 e^{2 \sqrt{g_2} (b_{1}( \tilde{\textit{t}} ) +b_{2}( \tilde{\textit{t}}) \tilde{x})})))). \end{aligned}$$

Set(4): If $g_{0}= g_{1}= g_{2}=g_{6}=0$, the following results are obtained: $(4.1) \ \hspace{0.1cm}\beta _{-1}=\beta _1=\beta _2=a_{32}=0,\ \beta _0=-\frac{a_{27}}{2 a_{33}},\hspace{0.1cm}\beta _{-2}=\frac{a_{22}}{4 a_{33} g_4},\ a_{28}=a_{27},\ a_{23}=\frac{a_{22} a_{27}}{2 a_{33}}, \hspace{0.1cm}a_{29}= -\frac{5}{2} a_{22},$ $\hspace{0.1cm}a_{31}=a_{30}=2 a_{33}.$ An examination of the system in set (4.1) indicates the existence of multiple solution types. Specifically, polynomial solutions arise under the conditions $g_{3}>0$ and $g_{4}a_{33}\ne 0$. Consequently, the characteristics of these solutions are predominantly influenced by the parameter values and their interdependencies, as discussed in the subsequent analysis of the system:

45 $$\begin{aligned} & \tilde{\theta }_{4.1}=\frac{a_{22} \left( g_3^3 (b_{1}( \tilde{\textit{t}} ) +b_{2}( \tilde{\textit{t}}) \tilde{x})^2-4 g_4\right) {}^2-32 a_{27} g_4^3}{64 a_{33} g_4^3}, \end{aligned}$$

46 $$\begin{aligned} & \tilde{u}_{4.2}=\frac{\xi \left( a_{22} \left( g_3^3 (b_{1}( \tilde{\textit{t}} ) +b_{2}( \tilde{\textit{t}}) \tilde{x})^2-4 g_4\right) {}^2-32 a_{27} g_4^3\right) }{64 a_{33} g_4^3}, \end{aligned}$$

according to the previous relations, the stress tensor is formulated as:

47 $$\begin{aligned} & \tilde{\tau }_{xx}= \frac{1}{4096 a_{33}^2 g_4^6} (256 a_{22} a_{33} b_2 g_4^3 g_3^3 \xi (b_{1}( \tilde{\textit{t}} ) +b_{2}( \tilde{\textit{t}}) \tilde{x}) (g_3^3 (b_{1}( \tilde{\textit{t}} ) +b_{2}( \tilde{\textit{t}}) \tilde{x})^2-4 g_4)-64 a_{33} g_4^3 l_1 (a_{22} (g_3^3 (b_{1}( \tilde{\textit{t}} ) \nonumber \\ & +b_{2}( \tilde{\textit{t}}) \tilde{x})^2-4 g_4){}^2-32 a_{27} g_4^3)-4 a_{22} a_{24} g_3^3 \xi (b_{1}( \tilde{\textit{t}} ) +b_{2}( \tilde{\textit{t}}) \tilde{x}) (g_3^3 (b_{1}( \tilde{\textit{t}} ) +b_{2}( \tilde{\textit{t}}) \tilde{x})^2-4 g_4) (a_{22} (g_3^3 (b_{1}( \tilde{\textit{t}} ) +b_{2}( \tilde{\textit{t}}) \tilde{x})^2\nonumber \\ & -4 g_4)^2-32 a_{27} g_4^3)+a_{25}(a_{22} (g_3^3 (b_{1}( \tilde{\textit{t}} ) +b_{2}( \tilde{\textit{t}}) \tilde{x})^2-4 g_4)^2-32 a_{27} g_4^3)^2). \end{aligned}$$

Set(5): If $g_{0}= g_{1}=g_{6}=0$, the following results are obtained: $(5.1)\hspace{0.1cm}\beta _{-1}=\beta _{-2}=0,\hspace{0.3cm}\beta _1=-\frac{9 \left( 9 a_{23} a_{31}^2+4 \left( 2 a_{27}^2+5 a_{28} a_{27}+3 a_{28}^2\right) a_{32}\right) g_3}{4 \left( 2 a_{27}+3 a_{28}\right) a_{32}^2},\hspace{0.3cm}\beta _0=-\frac{9 a_{23} a_{31}}{4 a_{27} a_{32}+6 a_{28} a_{32}}\hspace{0.3cm}a_{29}=-\frac{9 a_{23} a_{31}}{4 a_{27}+6 a_{28}},$ $\hspace{0.3cm}a_{30}=-a_{33},\hspace{0.3cm}\beta _2=\frac{81 a_{31} \left( 9 a_{23} a_{31}^2+4 \left( 2 a_{27}^2+5 a_{28} a_{27}+3 a_{28}^2\right) a_{32}\right) ^2 g_3^2}{64 \left( 2 a_{27}+3 a_{28}\right) ^3 a_{32}^4},\hspace{0.3cm}a_{22}=\frac{2 \left( 2 a_{27}+3 a_{28}\right) a_{32}}{9 a_{31}}.$ An examination of the system in set (5.1) indicates the existence of multiple solution types. Specifically, dark soliton solutions arise under the conditions $g_{2}>0$ , $g_{32}\ne 0$ and $2 a_{27}+3 a_{28}\ne 0$. Consequently, the characteristics of these solutions are predominantly influenced by the parameter values and their interdependencies, as discussed in the subsequent analysis of the system:

48 $$\begin{aligned} & \tilde{\theta }_{5.1}=\frac{81 a_{31} \left( 9 a_{23} a_{31}^2+4 \left( 2 a_{27}^2+5 a_{28} a_{27}+3 a_{28}^2\right) a_{32}\right) ^2 g_2^2 \left( \tanh \left( \frac{\sqrt{g_2} (b_{1}( \tilde{\textit{t}} ) +b_{2}( \tilde{\textit{t}}) \tilde{x})}{2}\right) +1\right) ^2}{64 \left( 2 a_{27}+3 a_{28}\right) ^3 a_{32}^4}\nonumber \\ & +\frac{9 \left( 9 a_{23} a_{31}^2+4 \left( 2 a_{27}^2+5 a_{28} a_{27}+3 a_{28}^2\right) a_{32}\right) g_2 \left( \tanh \left( \frac{\sqrt{g_2} (b_{1}( \tilde{\textit{t}} ) +b_{2}( \tilde{\textit{t}}) \tilde{x})}{2}\right) +1\right) }{4 \left( 2 a_{27}+3 a_{28}\right) a_{32}^2}-\frac{9 a_{23} a_{31}}{4 a_{27} a_{32}+6 a_{28} a_{32}}, \end{aligned}$$

49 $$\begin{aligned} & \tilde{u}_{5.1}=\xi (\frac{81 a_{31} \left( 9 a_{23} a_{31}^2+4 \left( 2 a_{27}^2+5 a_{28} a_{27}+3 a_{28}^2\right) a_{32}\right) {}^2 g_2^2 \left( \tanh \left( \frac{\sqrt{g_2} (b_{1}( \tilde{\textit{t}} ) +b_{2}( \tilde{\textit{t}}) \tilde{x})}{2}\right) +1\right) ^2}{64 \left( 2 a_{27}+3 a_{28}\right) ^3 a_{32}^4}\nonumber \\ & +\frac{9 \left( 9 a_{23} a_{31}^2+4 \left( 2 a_{27}^2+5 a_{28} a_{27}+3 a_{28}^2\right) a_{32}\right) g_2 \left( \tanh \left( \frac{\sqrt{g_2} (b_{1}( \tilde{\textit{t}} ) +b_{2}( \tilde{\textit{t}}) \tilde{x})}{2}\right) +1\right) }{4 \left( 2 a_{27}+3 a_{28}\right) a_{32}^2}-\frac{9 a_{23} a_{31}}{4 a_{27} a_{32}+6 a_{28} a_{32}}, \end{aligned}$$

according to the previous relations, the stress tensor is formulated as:

50 $$\begin{aligned} & \tilde{\tau }_{\text {xx}}=\frac{1}{64 \left( 2 a_{27}+3 a_{28}\right) ^3 a_{32}^4}(9 g_2^{3/2} \xi \nabla b_2 \text {sech}^2\left( \frac{\sqrt{g_2} (b_{1}( \tilde{\textit{t}} ) +b_{2}( \tilde{\textit{t}}) \tilde{x})}{2}\right) (9 a_{31} \nabla g_2 \left( \tanh \left( \frac{\sqrt{g_2} (b_{1}( \tilde{\textit{t}} ) +b_{2}( \tilde{\textit{t}}) \tilde{x})}{2}\right) +1\right) \nonumber \\ & +8 \left( 2 a_{27}+3 a_{28}\right) ^2 a_{32}^2)-l_1(-\frac{9 a_{23} a_{31}}{4 a_{27} a_{32}+6 a_{28} a_{32}}+\frac{9 \nabla g_2 \left( \tanh \left( \frac{\sqrt{g_2} (b_{1}( \tilde{\textit{t}} ) +b_{2}( \tilde{\textit{t}}) \tilde{x})}{2}\right) +1\right) }{4 \left( 2 a_{27}+3 a_{28}\right) a_{32}^2}\nonumber \\ & +\frac{81a_{31}\nabla ^2g_2^2\left( \tanh \left( \frac{\sqrt{g_2} (b_{1}( \tilde{\textit{t}} ) +b_{2}( \tilde{\textit{t}}) \tilde{x})}{2}\right) +1\right) ^2}{64 \left( 2 a_{27}+3 a_{28}\right) ^3 a_{32}^4})-\frac{9}{64 \left( 2 a_{27}+3 a_{28}\right) ^3 a_{32}^4}\xi \text {sech}^2\left( \frac{\sqrt{g_2} (b_{1}( \tilde{\textit{t}} ) +b_{2}( \tilde{\textit{t}}) \tilde{x})}{2}\right) a_{24}\nabla \nonumber \\ & g_2^{3/2} a_{31} \nabla g_2 \left( \tanh \left( \frac{\sqrt{g_2} (b_{1}( \tilde{\textit{t}} ) +b_{2}( \tilde{\textit{t}}) \tilde{x})}{2}\right) +1\right) +8 \left( 2 a_{27}+3 a_{28}\right) ^2 a_{32}^2(-\frac{9 a_{23} a_{31}}{4 a_{27} a_{32}+6 a_{28} a_{32}}\nonumber \\ & +\frac{9 \nabla g_2 \left( \tanh \left( \frac{\sqrt{g_2} (b_{1}( \tilde{\textit{t}} ) +b_{2}( \tilde{\textit{t}}) \tilde{x})}{2}\right) +1\right) }{4 \left( 2 a_{27}+3 a_{28}\right) a_{32}^2}+\frac{81a_{31}\nabla ^2 g_2^2\left( \tanh \left( \frac{\sqrt{g_2} (b_{1}( \tilde{\textit{t}} ) +b_{2}( \tilde{\textit{t}}) \tilde{x})}{2}\right) +1\right) ^2}{64 \left( 2 a_{27}+3 a_{28}\right) ^3 a_{32}^4}), \end{aligned}$$

where $$\nabla =9a_{23}a_{31}^2+4 \left( 2 a_{27}^2+5 a_{28} a_{27}+3 a_{28}^2\right) a_{32}$$.

Set(6): If $g_0=g_1=g_6=0$, the following results are obtained: $(6.1) \hspace{0.1cm}\beta _{-1}=\beta _{-2}=0,\hspace{0.3cm}\beta _1=\frac{3 \sqrt{18 a_{23} a_{31}^3+8 \left( 2 a_{27}^2+5 a_{28} a_{27}+3 a_{28}^2\right) a_{32} a_{31}} \sqrt{-g_4}}{a_{31} a_{32}},\hspace{0.3cm}\beta _0=-\frac{9 a_{23} a_{31}}{4 a_{27} a_{32}+6 a_{28} a_{32}},\hspace{0.3cm}a_{29}=-\frac{9 a_{23} a_{31}}{4 a_{27}+6 a_{28}},$

$\beta _2=-\frac{9 \left( 9 a_{23} a_{31}^2+4 \left( 2 a_{27}^2+5 a_{28} a_{27}+3 a_{28}^2\right) a_{32}\right) g_4}{2 \left( 2 a_{27}+3 a_{28}\right) a_{32}^2}, \hspace{0.3cm}a_{30}=-a_{33},\hspace{0.3cm}a_{22}=\frac{2 \left( 2 a_{27}+3 a_{28}\right) a_{32}}{9 a_{31}}.$ An examination of the system in set (6.1) indicates the existence of multiple solution types. Specifically, combo bright dark soliton solutions arise under the conditions $g_{2}g_{4}>0$ and $g_{32} (2 a_{27}+3 a_{28}) \ne 0$. Consequently, the characteristics of these solutions are predominantly influenced by the parameter values and their interdependencies, as discussed in the subsequent analysis of the system:

51 $$\begin{aligned} & \tilde{\theta }_{6.1}=-\frac{9 \left( 9 a_{23} a_{31}^2+4 \left( 2 a_{27}^2+5 a_{28} a_{27}+3 a_{28}^2\right) a_{32}\right) g_2^2 g_4 \text {sech}^4\left( \frac{\sqrt{g_2} (b_{1}( \tilde{\textit{t}} ) +b_{2}( \tilde{\textit{t}}) \tilde{x})}{2}\right) }{2 \left( 2 a_{27}+3 a_{28}\right) a_{32}^2 \left( g_3-2 \sqrt{g_2 g_4} \tanh \left( \frac{\sqrt{g_2} (b_{1}( \tilde{\textit{t}} ) +b_{2}( \tilde{\textit{t}}) \tilde{x})}{2}\right) \right) ^2}\nonumber \\ & -\frac{3 \sqrt{18 a_{23} a_{31}^3+8 \left( 2 a_{27}^2+5 a_{28} a_{27}+3 a_{28}^2\right) a_{32} a_{31}} g_2 \sqrt{-g_4} \text {sech}^2\left( \frac{\sqrt{g_2} (b_{1}( \tilde{\textit{t}} ) +b_{2}( \tilde{\textit{t}}) \tilde{x})}{2}\right) }{a_{31} a_{32} \left( g_3-2 \sqrt{g_2 g_4} \tanh \left( \frac{\sqrt{g_2} (b_{1}( \tilde{\textit{t}} ) +b_{2}( \tilde{\textit{t}}) \tilde{x})}{2}\right) \right) }-\frac{9 a_{23} a_{31}}{4 a_{27} a_{32}+6 a_{28} a_{32}},\nonumber \\ \end{aligned}$$

52 $$\begin{aligned} & \tilde{u}_{6.1}=\xi (-\frac{9 \left( 9 a_{23} a_{31}^2+4 \left( 2 a_{27}^2+5 a_{28} a_{27}+3 a_{28}^2\right) a_{32}\right) g_2^2 g_4 \text {sech}^4\left( \frac{\sqrt{g_2} (b_{1}( \tilde{\textit{t}} ) +b_{2}( \tilde{\textit{t}}) \tilde{x})}{2}\right) }{2 \left( 2 a_{27}+3 a_{28}\right) a_{32}^2 \left( g_3-2 \sqrt{g_2 g_4} \tanh \left( \frac{\sqrt{g_2} (b_{1}( \tilde{\textit{t}} ) +b_{2}( \tilde{\textit{t}}) \tilde{x})}{2}\right) \right) ^2}\nonumber \\ & -\frac{3 \sqrt{18 a_{23} a_{31}^3+8 \left( 2 a_{27}^2+5 a_{28} a_{27}+3 a_{28}^2\right) a_{32} a_{31}} g_2 \sqrt{-g_4} \text {sech}^2\left( \frac{\sqrt{g_2} (b_{1}( \tilde{\textit{t}} ) +b_{2}( \tilde{\textit{t}}) \tilde{x})}{2}\right) }{a_{31} a_{32} \left( g_3-2 \sqrt{g_2 g_4} \tanh \left( \frac{\sqrt{g_2} (b_{1}( \tilde{\textit{t}} ) +b_{2}( \tilde{\textit{t}}) \tilde{x})}{2}\right) \right) }-\frac{9 a_{23} a_{31}}{4 a_{27} a_{32}+6 a_{28} a_{32}}), \end{aligned}$$

according to the previous relations, the stress tensor is formulated as:

$$\begin{aligned} & \tilde{\tau }_{xx}=a_{25} (-\frac{9 \left( 9 a_{23} a_{31}^2+4 \left( 2 a_{27}^2+5 a_{28} a_{27}+3 a_{28}^2\right) a_{32}\right) g_2^2 g_4 \text {sech}^4\left( \frac{(b_{1}( \tilde{\textit{t}} ) +b_{2}( \tilde{\textit{t}}) \tilde{x}) \sqrt{g_2}}{2}\right) }{2 \left( 2 a_{27}+3 a_{28}\right) a_{32}^2 \left( g_3-2 \sqrt{g_2 g_4} \tanh \left( \frac{(b_{1}( \tilde{\textit{t}} ) +b_{2}( \tilde{\textit{t}}) \tilde{x}) \sqrt{g_2}}{2}\right) \right) {}^2}\nonumber \\ & -\frac{3 \sqrt{18 a_{23} a_{31}^3+8 \left( 2 a_{27}^2+5 a_{28} a_{27}+3 a_{28}^2\right) a_{32} a_{31}} g_2 \sqrt{-g_4} \text {sech}^2\left( \frac{(b_{1}( \tilde{\textit{t}} ) +b_{2}( \tilde{\textit{t}}) \tilde{x}) \sqrt{g_2}}{2}\right) }{a_{31} a_{32} \left( g_3-2 \sqrt{g_2 g_4} \tanh \left( \frac{(b_{1}( \tilde{\textit{t}} ) +b_{2}( \tilde{\textit{t}}) \tilde{x}) \sqrt{g_2}}{2}\right) \right) }-\frac{9 a_{23} a_{31}}{4 a_{27} a_{32}+6 a_{28} a_{32}}){}^2\nonumber \\ & -l_1 (-\frac{9 \left( 9 a_{23} a_{31}^2+4 \left( 2 a_{27}^2+5 a_{28} a_{27}+3 a_{28}^2\right) a_{32}\right) g_2^2 g_4 \text {sech}^4\left( \frac{(b_{1}( \tilde{\textit{t}} ) +b_{2}( \tilde{\textit{t}}) \tilde{x}) \sqrt{g_2}}{2}\right) }{2 \left( 2 a_{27}+3 a_{28}\right) a_{32}^2 \left( g_3-2 \sqrt{g_2 g_4} \tanh \left( \frac{(b_{1}( \tilde{\textit{t}} ) +b_{2}( \tilde{\textit{t}}) \tilde{x}) \sqrt{g_2}}{2}\right) \right) {}^2}\nonumber \\ & -\frac{3 \sqrt{18 a_{23} a_{31}^3+8 \left( 2 a_{27}^2+5 a_{28} a_{27}+3 a_{28}^2\right) a_{32} a_{31}} g_2 \sqrt{-g_4} \text {sech}^2\left( \frac{(b_{1}( \tilde{\textit{t}} ) +b_{2}( \tilde{\textit{t}}) \tilde{x}) \sqrt{g_2}}{2}\right) }{a_{31} a_{32} \left( g_3-2 \sqrt{g_2 g_4} \tanh \left( \frac{(b_{1}( \tilde{\textit{t}} ) +b_{2}( \tilde{\textit{t}}) \tilde{x}) \sqrt{g_2}}{2}\right) \right) }-\frac{9 a_{23} a_{31}}{4 a_{27} a_{32}+6 a_{28} a_{32}})\nonumber \\ & -\xi a_{24} (\frac{3 \hspace{0.1cm}\text {sech}^2\left( \frac{(b_{1}( \tilde{\textit{t}} ) +b_{2}( \tilde{\textit{t}}) \tilde{x}) \sqrt{g_2}}{2}\right) \sqrt{18 a_{23} a_{31}^3+8 \left( 2 a_{27}^2+5 a_{28} a_{27}+3 a_{28}^2\right) a_{32} a_{31}} \sqrt{-g_4} \tanh \left( \frac{(b_{1}( \tilde{\textit{t}} ) +b_{2}( \tilde{\textit{t}}) \tilde{x}) \sqrt{g_2}}{2}\right) g_2^{3/2}}{a_{31} a_{32} \left( g_3-2 \sqrt{g_2 g_4} \tanh \left( \frac{(b_{1}( \tilde{\textit{t}} ) +b_{2}( \tilde{\textit{t}}) \tilde{x}) \sqrt{g_2}}{2}\right) \right) }\nonumber \\ & -\frac{3 \hspace{0.1cm}\text {sech}^4\left( \frac{(b_{1}( \tilde{\textit{t}} ) +b_{2}( \tilde{\textit{t}}) \tilde{x}) \sqrt{g_2}}{2}\right) \sqrt{18 a_{23} a_{31}^3+8 \left( 2 a_{27}^2+5 a_{28} a_{27}+3 a_{28}^2\right) a_{32} a_{31}} \sqrt{-g_4} \sqrt{g_2 g_4} g_2^{3/2}}{a_{31} a_{32} \left( g_3-2 \sqrt{g_2 g_4} \tanh \left( \frac{(b_{1}( \tilde{\textit{t}} ) +b_{2}( \tilde{\textit{t}}) \tilde{x}) \sqrt{g_2}}{2}\right) \right) {}^2}\nonumber \\ & +\frac{9\hspace{0.1cm} \text {sech}^4\left( \frac{(b_{1}( \tilde{\textit{t}} ) +b_{2}( \tilde{\textit{t}}) \tilde{x}) \sqrt{g_2}}{2}\right) \left( 9 a_{23} a_{31}^2+4 \left( 2 a_{27}^2+5 a_{28} a_{27}+3 a_{28}^2\right) a_{32}\right) g_4 \tanh \left( \frac{(b_{1}( \tilde{\textit{t}} ) +b_{2}( \tilde{\textit{t}}) \tilde{x}) \sqrt{g_2}}{2}\right) g_2^{5/2}}{\left( 2 a_{27}+3 a_{28}\right) a_{32}^2 \left( g_3-2 \sqrt{g_2 g_4} \tanh \left( \frac{(b_{1}( \tilde{\textit{t}} ) +b_{2}( \tilde{\textit{t}}) \tilde{x}) \sqrt{g_2}}{2}\right) \right) {}^2}\nonumber \\ & -\frac{9\hspace{0.1cm} \text {sech}^6\left( \frac{(b_{1}( \tilde{\textit{t}} ) +b_{2}( \tilde{\textit{t}}) \tilde{x}) \sqrt{g_2}}{2}\right) \left( 9 a_{23} a_{31}^2+4 \left( 2 a_{27}^2+5 a_{28} a_{27}+3 a_{28}^2\right) a_{32}\right) g_4 \sqrt{g_2 g_4} g_2^{5/2}}{\left( 2 a_{27}+3 a_{28}\right) a_{32}^2 \left( g_3-2 \sqrt{g_2 g_4} \tanh \left( \frac{(b_{1}( \tilde{\textit{t}} ) +b_{2}( \tilde{\textit{t}}) \tilde{x}) \sqrt{g_2}}{2}\right) \right) {}^3}) \nonumber \\ & \times (-\frac{9 \left( 9 a_{23} a_{31}^2+4 \left( 2 a_{27}^2+5 a_{28} a_{27}+3 a_{28}^2\right) a_{32}\right) g_2^2 g_4 \text {sech}^4\left( \frac{(b_{1}( \tilde{\textit{t}} ) +b_{2}( \tilde{\textit{t}}) \tilde{x}) \sqrt{g_2}}{2}\right) }{2 \left( 2 a_{27}+3 a_{28}\right) a_{32}^2 \left( g_3-2 \sqrt{g_2 g_4} \tanh \left( \frac{(b_{1}( \tilde{\textit{t}} ) +b_{2}( \tilde{\textit{t}}) \tilde{x}) \sqrt{g_2}}{2}\right) \right) {}^2}\nonumber \\ & -\frac{3 \sqrt{18 a_{23} a_{31}^3+8 \left( 2 a_{27}^2+5 a_{28} a_{27}+3 a_{28}^2\right) a_{32} a_{31}} g_2 \sqrt{-g_4} \text {sech}^2\left( \frac{(b_{1}( \tilde{\textit{t}} ) +b_{2}( \tilde{\textit{t}}) \tilde{x}) \sqrt{g_2}}{2}\right) }{a_{31} a_{32} \left( g_3-2 \sqrt{g_2 g_4} \tanh \left( \frac{(b_{1}( \tilde{\textit{t}} ) +b_{2}( \tilde{\textit{t}}) \tilde{x}) \sqrt{g_2}}{2}\right) \right) }-\frac{9 a_{23} a_{31}}{4 a_{27} a_{32}+6 a_{28} a_{32}})\nonumber \\ & +\xi b_2 (\frac{3 \text {sech}^2\left( \frac{(b_{1}( \tilde{\textit{t}} ) +b_{2}( \tilde{\textit{t}}) \tilde{x}) \sqrt{g_2}}{2}\right) \sqrt{18 a_{23} a_{31}^3+8 \left( 2 a_{27}^2+5 a_{28} a_{27}+3 a_{28}^2\right) a_{32} a_{31}} \sqrt{-g_4} \tanh \left( \frac{(b_{1}( \tilde{\textit{t}} ) +b_{2}( \tilde{\textit{t}}) \tilde{x}) \sqrt{g_2}}{2}\right) g_2^{3/2}}{a_{31} a_{32} \left( g_3-2 \sqrt{g_2 g_4} \tanh \left( \frac{(b_{1}( \tilde{\textit{t}} ) +b_{2}( \tilde{\textit{t}}) \tilde{x}) \sqrt{g_2}}{2}\right) \right) }\nonumber \\ & -\frac{3\hspace{0.1cm} \text {sech}^4\left( \frac{(b_{1}( \tilde{\textit{t}} ) +b_{2}( \tilde{\textit{t}}) \tilde{x}) \sqrt{g_2}}{2}\right) \sqrt{18 a_{23} a_{31}^3+8 \left( 2 a_{27}^2+5 a_{28} a_{27}+3 a_{28}^2\right) a_{32} a_{31}} \sqrt{-g_4} \sqrt{g_2 g_4} g_2^{3/2}}{a_{31} a_{32} \left( g_3-2 \sqrt{g_2 g_4} \tanh \left( \frac{(b_{1}( \tilde{\textit{t}} ) +b_{2}( \tilde{\textit{t}}) \tilde{x}) \sqrt{g_2}}{2}\right) \right) {}^2}\nonumber \\ \end{aligned}$$

53 $$\begin{aligned} & +\frac{9 \hspace{0.1cm} \text {sech}^4\left( \frac{(b_{1}( \tilde{\textit{t}} ) +b_{2}( \tilde{\textit{t}}) \tilde{x}) \sqrt{g_2}}{2}\right) \left( 9 a_{23} a_{31}^2+4 \left( 2 a_{27}^2+5 a_{28} a_{27}+3 a_{28}^2\right) a_{32}\right) g_4 \tanh \left( \frac{(b_{1}( \tilde{\textit{t}} ) +b_{2}( \tilde{\textit{t}}) \tilde{x}) \sqrt{g_2}}{2}\right) g_2^{5/2}}{\left( 2 a_{27}+3 a_{28}\right) a_{32}^2 \left( g_3-2 \sqrt{g_2 g_4} \tanh \left( \frac{(b_{1}( \tilde{\textit{t}} ) +b_{2}( \tilde{\textit{t}}) \tilde{x}) \sqrt{g_2}}{2}\right) \right) ^2}\nonumber \\ & -\frac{9 \hspace{0.1cm} \text {sech}^6\left( \frac{(b_{1}( \tilde{\textit{t}} ) +b_{2}( \tilde{\textit{t}}) \tilde{x}) \sqrt{g_2}}{2}\right) \left( 9 a_{23} a_{31}^2+4 \left( 2 a_{27}^2+5 a_{28} a_{27}+3 a_{28}^2\right) a_{32}\right) g_4 \sqrt{g_2 g_4} g_2^{5/2}}{\left( 2 a_{27}+3 a_{28}\right) a_{32}^2 \left( g_3-2 \sqrt{g_2 g_4} \tanh \left( \frac{(b_{1}( \tilde{\textit{t}} ) +b_{2}( \tilde{\textit{t}}) \tilde{x}) \sqrt{g_2}}{2}\right) \right) ^3}). \end{aligned}$$

Set(7): If $g_{1}= g_{3}=g_{6}=0$, the following results are obtained: $(7.1) \hspace{0.1cm}\beta _0=\frac{\sqrt{a_{30} a_{23} \left( 3 a_{30}+4 a_{33}\right) }}{a_{30} \sqrt{6 a_{32}}},\hspace{0.3cm}\beta _{-1}=g_0=\beta _1=\beta _2=0,\hspace{0.3cm}\beta _{-2}=-\frac{\sqrt{\frac{2 a_{23} a_{32}}{3 a_{30} \left( 3 a_{30}+4 a_{33}\right) }}}{g_4},\hspace{0.3cm}g_2=-\frac{a_{32}}{3 a_{30}+4 a_{33}},$

$a_{29}=5 a_{23} a_{30} a_{32} \sqrt{\frac{2}{a_{30} \left( 3 a_{30}+4 a_{33}\right) }},\hspace{0.3cm}a_{31}=\frac{a_{30}}{2},\hspace{0.3cm}a_{22}=\frac{4 a_{28} a_{32}}{3 a_{30}+4 a_{33}}-2 a_{30} \sqrt{\frac{2 a_{23} a_{32}}{3 a_{30} \left( 3 a_{30}+4 a_{33}\right) }}, \ a_{27}=-\sqrt{\frac{a_{30} a_{23} \left( 3 a_{30}+4 a_{33}\right) }{6 a_{32}}}.$ Examining the set (7.1) leads to multiple solution types for the given model: (7.1. 1) Singular solitons emerge under the conditions $g_{4}\ne 0$, $g_{32}\ne 0$ and $6 a_{32} a_{30} \left( 3 a_{30}+4 a_{33}\right) \ne 0$. Consequently, the behavior of these solutions is fundamentally governed by the interplay and specific values of these parameters, as elaborated in the subsequent analysis of the system:

54 $$\begin{aligned} & \bar{\theta }_{7.1}=\sqrt{\frac{a_{23}}{6 a_{32} a_{30} \left( 3 a_{30}+4 a_{33}\right) }}\left( \frac{ \left( 3 a_{30}+4 a_{33}\right) g_4-2 a_{32} \coth ^2(b_{1}( \tilde{\textit{t}} ) +b_{2}( \tilde{\textit{t}}) \tilde{x})}{g_4}\right) , \end{aligned}$$

55 $$\begin{aligned} & \bar{u}_{7.1}=\xi \sqrt{\frac{a_{23}}{6 a_{32} a_{30} \left( 3 a_{30}+4 a_{33}\right) }}\left( \frac{ \left( 3 a_{30}+4 a_{33}\right) g_4-2 a_{32} \coth ^2(b_{1}( \tilde{\textit{t}} ) +b_{2}( \tilde{\textit{t}}) \tilde{x})}{g_4}\right) , \end{aligned}$$

according to the previous relations, the stress tensor is formulated as:

56 $$\begin{aligned} & \bar{\tau }_{xx}=\frac{1}{6 g_4^2}(4 \sqrt{6} \sqrt{\frac{a_{23}}{a_{30} a_{32} \left( 3 a_{30}+4 a_{33}\right) }} a_{32} b_2 g_4 \xi \coth (b_{1}( \tilde{\textit{t}} ) +b_{2}( \tilde{\textit{t}}) \tilde{x}) \text {csch}^2(b_{1}( \tilde{\textit{t}} ) +b_{2}( \tilde{\textit{t}}) \tilde{x})\nonumber \\ & -\sqrt{6} \sqrt{\frac{a_{23}}{a_{30} a_{32} \left( 3 a_{30}+4 a_{33}\right) }} g_4 l_1 \left( \left( 3 a_{30}+4 a_{33}\right) g_4-2 a_{32} \coth ^2(b_{1}( \tilde{\textit{t}} ) +b_{2}( \tilde{\textit{t}}) \tilde{x})\right) \nonumber \\ & -\frac{4 a_{23} a_{24} \xi \coth (b_{1}( \tilde{\textit{t}} ) +b_{2}( \tilde{\textit{t}}) \tilde{x}) \text {csch}^2(b_{1}( \tilde{\textit{t}} ) +b_{2}( \tilde{\textit{t}}) \tilde{x}) \left( \left( 3 a_{30}+4 a_{33}\right) g_4-2 a_{32} \coth ^2(b_{1}( \tilde{\textit{t}} ) +b_{2}( \tilde{\textit{t}}) \tilde{x})\right) }{a_{30} \left( 3 a_{30}+4 a_{33}\right) }\nonumber \\ & +\frac{a_{23} a_{25} \left( \left( 3 a_{30}+4 a_{33}\right) g_4-2 a_{32} \coth ^2(b_{1}( \tilde{\textit{t}} ) +b_{2}( \tilde{\textit{t}}) \tilde{x})\right) ^2}{a_{30} a_{32} \left( 3 a_{30}+4 a_{33}\right) }). \end{aligned}$$

(7.1. 2) Dark solitons emerge under the conditions $g_{4}\ne 0$, $a_{23}a_{32}\ne 0$, $g_{30}\ne 0$ and $3 a_{30}^2+4 a_{33} a_{30} \ne 0$. Consequently, the behavior of these solutions is fundamentally governed by the interplay and specific values of these parameters, as elaborated in the subsequent analysis of the system:

57 $$\begin{aligned} & \bar{\theta }_{7.2}=\frac{1}{\sqrt{6}}\left( \frac{\sqrt{\frac{a_{23} a_{30} \left( 3 a_{30}+4 a_{33}\right) }{a_{32}}}}{a_{30}}-\frac{\sqrt{\frac{a_{23} a_{32}}{3 a_{30}^2+4 a_{33} a_{30}}} \tanh ^2(b_{1}( \tilde{\textit{t}} ) +b_{2}( \tilde{\textit{t}}) \tilde{x})}{g_4}\right) , \end{aligned}$$

58 $$\begin{aligned} & \bar{u}_{7.2}=\frac{\xi }{\sqrt{6}}\left( \frac{\sqrt{\frac{a_{23} a_{30} \left( 3 a_{30}+4 a_{33}\right) }{a_{32}}}}{a_{30}}-\frac{\sqrt{\frac{a_{23} a_{32}}{3 a_{30}^2+4 a_{33} a_{30}}} \tanh ^2(b_{1}( \tilde{\textit{t}} ) +b_{2}( \tilde{\textit{t}}) \tilde{x})}{g_4}\right) , \end{aligned}$$

according to the previous relations, the stress tensor is formulated as:

59 $$\begin{aligned} & \bar{\tau }_{xx}=\frac{1}{6 a_{30} a_{32} g_4^2}(\sqrt{a_{32}} g_4 (\sqrt{\frac{a_{23} a_{32}}{3 a_{30}^2+4 a_{33} a_{30}}} \tanh (b_{1}( \tilde{\textit{t}} ) +b_{2}( \tilde{\textit{t}}) \tilde{x}) \text {sech}^2(b_{1}( \tilde{\textit{t}} ) +b_{2}( \tilde{\textit{t}}) \tilde{x}) (\sqrt{6} \sqrt{a_{32}} a_{30}\nonumber \\ & \times (l_1 \sinh (2 (b_{1}( \tilde{\textit{t}} ) +b_{2}( \tilde{\textit{t}}) \tilde{x}))-4 b_2 \xi )+4 \sqrt{a_{23} a_{30} (3 a_{30}+4 a_{33})} a_{24} \xi -2 a_{25} \sqrt{a_{23} a_{30} \left( 3 a_{30}+4 a_{33}\right) }\nonumber \\ & \times \sinh (2 (b_{1}( \tilde{\textit{t}} ) +b_{2}( \tilde{\textit{t}}) \tilde{x})))-\sqrt{6} \sqrt{a_{23} a_{30} \left( 3 a_{30}+4 a_{33}\right) } g_4 L_1)\nonumber \\ & +\frac{1}{3 a_{30}+4 a_{33}}(a_{23} (a_{25} \left( 9 a_{30}^2 g_4^2+16 a_{33}^2 g_4^2+24 a_{30} a_{33} g_4^2+4 a_{32}^2 \tanh ^4(b_{1}( \tilde{\textit{t}} ) +b_{2}( \tilde{\textit{t}}) \tilde{x})\right) -8 a_{24} a_{32}^2 \xi \nonumber \\ & \times \tanh ^3(b_{1}( \tilde{\textit{t}} ) +b_{2}( \tilde{\textit{t}}) \tilde{x}) \text {sech}^2(b_{1}( \tilde{\textit{t}} ) +b_{2}( \tilde{\textit{t}}) \tilde{x}))). \end{aligned}$$

(7.1. 3) Hyperbolic solutions emerge under the conditions $g_{4}\ne 0$, $a_{23}a_{32}\ne 0$, $g_{30}\ne 0$ and $3 a_{30}^2+4 a_{33} a_{30} \ne 0$. Consequently, the behavior of these solutions is fundamentally governed by the interplay and specific values of these parameters, as elaborated in the subsequent analysis of the system:

60 $$\begin{aligned} & \bar{\theta }_{7.3}=\frac{1}{\sqrt{6}}\left( \frac{\sqrt{\frac{a_{23} a_{30} \left( 3 a_{30}+4 a_{33}\right) }{a_{32}}}}{a_{30}}-\frac{ \sqrt{\frac{a_{23} a_{32}}{3 a_{30}^2+4 a_{33} a_{30}}} \cosh ^2(b_{1}( \tilde{\textit{t}} ) +b_{2}( \tilde{\textit{t}}) \tilde{x})}{g_4}\right) , \end{aligned}$$

61 $$\begin{aligned} & \bar{u}_{7.3}= \frac{\xi }{\sqrt{6}}\left( \frac{\sqrt{\frac{a_{23} a_{30} \left( 3 a_{30}+4 a_{33}\right) }{a_{32}}}}{a_{30}}-\frac{ \sqrt{\frac{a_{23} a_{32}}{3 a_{30}^2+4 a_{33} a_{30}}} \cosh ^2(b_{1}( \tilde{\textit{t}} ) +b_{2}( \tilde{\textit{t}}) \tilde{x})}{g_4}\right) , \end{aligned}$$

according to the previous relations, the stress tensor is formulated as:

62 $$\begin{aligned} & \bar{\tau }_{xx}=-\frac{\sqrt{2} \sqrt{\frac{a_{23} a_{32}}{9 a_{30}^2+12 a_{33} a_{30}}} b_2 \xi \sinh (2 (b_{1}( \tilde{\textit{t}} ) +b_{2}( \tilde{\textit{t}}) \tilde{x}))}{g_4}-\frac{l_1 \left( \frac{\sqrt{a_{23} a_{30} \left( 3 a_{30}+4 a_{33}\right) }}{a_{30} \sqrt{a_{32}}}-\frac{ \sqrt{\frac{a_{23} a_{32}}{3 a_{30}^2+4 a_{33} a_{30}}} \cosh ^2(b_{1}( \tilde{\textit{t}} ) +b_{2}( \tilde{\textit{t}}) \tilde{x})}{g_4}\right) }{\sqrt{6}}\nonumber \\ & +\frac{a_{24} \xi \sinh (2 (b_{1}( \tilde{\textit{t}} ) +b_{2}( \tilde{\textit{t}}) \tilde{x})) \left( \sqrt{a_{23} a_{30} \left( 3 a_{30}+4 a_{33}\right) } \sqrt{\frac{a_{23} a_{32}}{3 a_{30}^2+4 a_{33} a_{30}}} g_4-\frac{2 a_{23} a_{32}^{3/2} \cosh ^2(b_{1}( \tilde{\textit{t}} ) +b_{2}( \tilde{\textit{t}}) \tilde{x})}{3 a_{30}+4 a_{33}}\right) }{3 a_{30} \sqrt{a_{32}} g_4^2}\nonumber \\ & +\frac{1}{6} a_{25} \left( \frac{\sqrt{a_{23} a_{30} \left( 3 a_{30}+4 a_{33}\right) }}{a_{30} \sqrt{a_{32}}}-\frac{ \sqrt{\frac{a_{23} a_{32}}{3 a_{30}^2+4 a_{33} a_{30}}} \cosh ^2(b_{1}( \tilde{\textit{t}} ) +b_{2}( \tilde{\textit{t}}) \tilde{x})}{g_4}\right) ^2. \end{aligned}$$

(7.1. 4) Bright soliton solutions emerge under the conditions $g_{4}\ne 0$, $a_{23}a_{32}\ne 0$, $g_{30}\ne 0$ and $3 a_{30}^2+4 a_{33} a_{30} \ne 0$. Consequently, the behavior of these solutions is fundamentally governed by the interplay and specific values of these parameters, as elaborated in the subsequent analysis of the system:

63 $$\begin{aligned} & \bar{\theta }_{7.4}=\frac{1}{\sqrt{6}}\left( \frac{\sqrt{\frac{a_{23} a_{30} \left( 3 a_{30}+4 a_{33}\right) }{a_{32}}}}{a_{30}}-\frac{ \sqrt{\frac{a_{23} a_{32}}{3 a_{30}^2+4 a_{33} a_{30}}} \text {sech}^2(b_{1}( \tilde{\textit{t}} ) +b_{2}( \tilde{\textit{t}}) \tilde{x})}{g_4}\right) , \end{aligned}$$

64 $$\begin{aligned} & \bar{u}_{7.4}=\frac{\xi }{\sqrt{6}}\left( \frac{\sqrt{\frac{a_{23} a_{30} \left( 3 a_{30}+4 a_{33}\right) }{a_{32}}}}{a_{30}}-\frac{ \sqrt{\frac{a_{23} a_{32}}{3 a_{30}^2+4 a_{33} a_{30}}} \text {sech}^2(b_{1}( \tilde{\textit{t}} ) +b_{2}( \tilde{\textit{t}}) \tilde{x})}{g_4}\right) , \end{aligned}$$

according to the previous relations, the stress tensor is formulated as:

65 $$\begin{aligned} & \bar{\tau }_{xx}=\frac{ \sqrt{2} \sqrt{\frac{a_{23} a_{32}}{9 a_{30}^2+12 a_{33} a_{30}}} b_2 \xi \tanh (b_{1}( \tilde{\textit{t}} ) +b_{2}( \tilde{\textit{t}}) \tilde{x}) \text {sech}^2(b_{1}( \tilde{\textit{t}} ) +b_{2}( \tilde{\textit{t}}) \tilde{x})}{g_4}\nonumber \\ & -\frac{l_1 \left( \frac{\sqrt{a_{23} a_{30} \left( 3 a_{30}+4 a_{33}\right) }}{a_{30} \sqrt{a_{32}}}-\frac{ \sqrt{\frac{a_{23} a_{32}}{3 a_{30}^2+4 a_{33} a_{30}}} \text {sech}^2(b_{1}( \tilde{\textit{t}} ) +b_{2}( \tilde{\textit{t}}) \tilde{x})}{g_4}\right) }{\sqrt{6}}\nonumber \\ & +\frac{2 a_{24} \xi \tanh (b_{1}( \tilde{\textit{t}} ) +b_{2}( \tilde{\textit{t}}) \tilde{x}) \text {sech}^2(b_{1}( \tilde{\textit{t}} ) +b_{2}( \tilde{\textit{t}}) \tilde{x}) \left( \frac{2 a_{23} a_{32}^{3/2} \text {sech}^2(b_{1}( \tilde{\textit{t}} ) +b_{2}( \tilde{\textit{t}}) \tilde{x})}{3 a_{30}+4 a_{33}}-\sqrt{a_{30} a_{32}} a_{23} g_4\right) }{3 a_{30} \sqrt{a_{32}} g_4^2}\nonumber \\ & +\frac{1}{6} a_{25} \left( \frac{\sqrt{a_{23} a_{30} \left( 3 a_{30}+4 a_{33}\right) }}{a_{30} \sqrt{a_{32}}}-\frac{ \sqrt{\frac{a_{23} a_{32}}{3 a_{30}^2+4 a_{33} a_{30}}} \text {sech}^2(b_{1}( \tilde{\textit{t}} ) +b_{2}( \tilde{\textit{t}}) \tilde{x})}{g_4}\right) ^2. \end{aligned}$$

## Discussion

In this section, two-dimensional graphical depictions of specific solutions are showcased. Copper is selected as the thermoelastic material in this study, with its physical constants assigned the following values^63^:

$$\begin{aligned} T_{0}= & 2.93\times 10^{2}K, \quad c_{e}=3.831\times 10^{2}J.kg^{-1}.K^{-1}, \quad \mathring{k}=368 w.m^{-1}.K^{-1}, \\ \mathring{\rho }= & 89.54\times 10^{2}kg.m^{-3}, \quad \mathring{\mu }=38.6\times 10^{9}N.m^{-2}, \quad \mathring{\lambda }=77.6\times 10^{9}N.m^{-2}, \\ \alpha _{t}= & 1.78\times 10^{-5}K^{-1}. \end{aligned}$$

along with the laser pulse parameters, are:

$$\begin{aligned} \gamma =0.5m^{-1}, \quad t_{0}=0.4P.S, \quad r=8\mu m. \end{aligned}$$

**Fig. 1:**
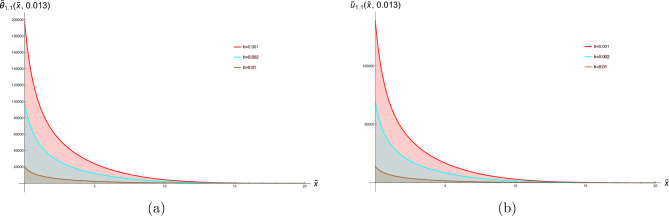
Diagrams in two dimensions illustrate the hyperbolic solutions obtained from Eqs. (30) and (31).

**Fig. 2:**
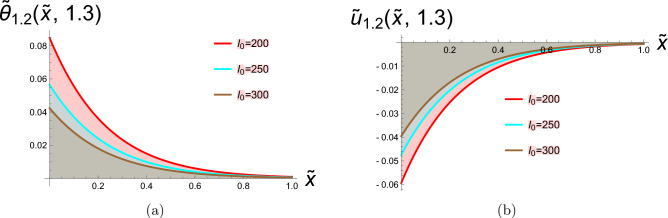
Diagrams in two dimensions illustrate the bright soliton solutions obtained from Eqs. (33) and (34).

**Fig. 3:**
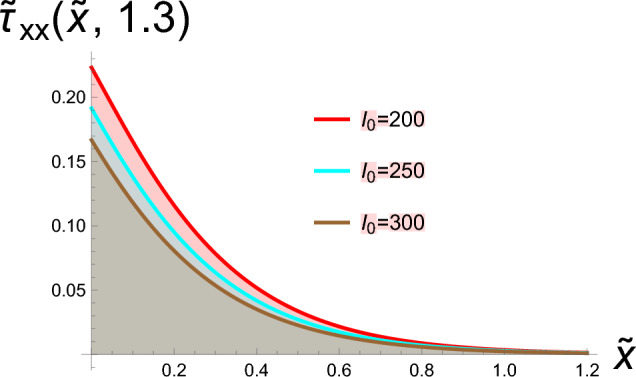
Diagrams in two dimensions illustrate the stress tensor for Eq. (35).

**Fig. 4:**
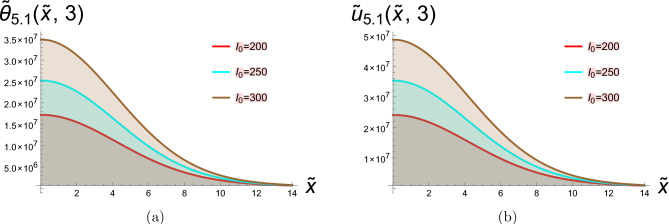
Diagrams in two dimensions illustrate the dark soliton solutions from Eqs. (48) and (49).

**Fig. 5:**
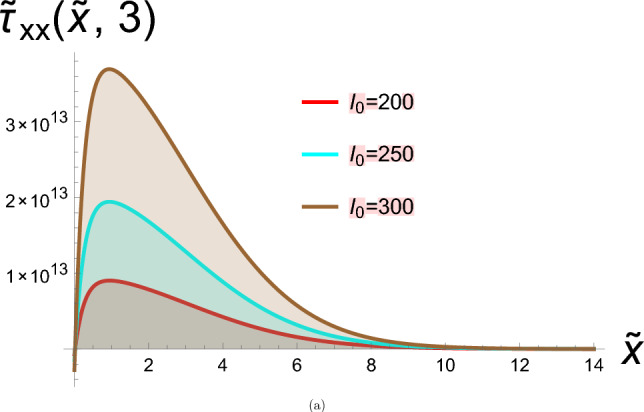
Diagrams in two dimensions illustrate the stress tensor for Eq. (50).

The physical configuration of the problem consists of a homogeneous, isotropic thermoelastic half-space occupying the region $x\ge 0$. The medium is initially at a uniform reference temperature and is assumed to be free from external disturbances. A laser pulse is applied at the boundary $x=0$, acting as a localized and time-dependent heat source that induces thermal and mechanical responses within the medium. Due to the symmetry and nature of the loading, all field variables depend only on the spatial coordinate *x* and time *t*. This simplified geometrical model captures the essential features of laser-induced thermoelastic behavior. To address the parametric rigor and provide a comprehensive analysis beyond pulse intensity ($I_0$), we systematically investigate the influence of laser pulse duration ($t_0$), material properties ($\mathring{\rho }, \mathring{\mu }, \mathring{\lambda }, \alpha _t$), and spatial beam characteristics ($r, \gamma$). This expanded parametric study allows for a deeper understanding of how different thermoelastic and laser parameters independently and interactively govern the system’s response. For the lower thermal load ($h = 0.001$), the discrepancy between the temperature-dependent and constant-property models is present but relatively modest. This suggests that isothermal property assumptions might provide a first-order approximation under mild heating conditions. However, as the thermal load intensifies to $h = 0.002$ and further to $h = 0.01$, the gap between the two curves widens considerably. The temperature-dependent model predicts a non-linear response that is not captured by the constant-property solution. This amplification of the difference is a direct mechanistic consequence of the material’s evolving properties: the decrease in stiffness (Young’s modulus) and changes in the thermal expansion coefficient at elevated temperatures lead to larger deformations and altered stress fields than those predicted by a model that fails to account for this softening effect. Therefore, the comparison in Fig. 1 depicts hyperbolic solutions from Eqs. (30) and (31), with parameters set to  $g_2=0.4,\hspace{0.2cm}g_4=2,\hspace{0.2cm}\xi =0.7,\hspace{0.2cm}b_1=\tilde{\textit{t}}, \ I_0=100, \text {and} \ \hspace{0.2cm}b _2=\tilde{\textit{t}}$, this figure conclusively highlights that the role of temperature-dependent material properties is not merely incremental but fundamental, especially under moderate to severe thermal loading. Assuming constant properties underestimates the actual thermoelastic response, which could have critical implications for the design and failure analysis of components operating in thermal environments. Physically, this implies that when material properties exhibit greater sensitivity to temperature variations, the heat conduction process becomes more effective in elevating local temperatures, thereby amplifying the thermal wave amplitude. Consequently, materials with stronger temperature-dependent characteristics undergo more pronounced mechanical deformation under the same thermal loading Fig. 2 depicts bright soliton solutions from Eqs. (33) and (34), with parameters set to  $g_2=4,\hspace{0.2cm}g_4=2,\hspace{0.2cm}\xi =-0.7,\hspace{0.2cm}b_1=\tilde{\textit{t}}, \text {and} \ \hspace{0.2cm}b _2=\tilde{\textit{t}}$. Figure 2a presents the spatial distribution of the dimensionless temperature $\tilde{\theta }$ versus the normalized spatial coordinate $\tilde{x}$ for three different laser pulse intensities. The curves reflect how a laser pulse introduces localized thermal energy into a thermoelastic medium. As the laser intensity $I_{0}$ increases the peak temperature rises and the thermal penetration extends slightly further into the material. This behavior is consistent with physical expectations, as laser pulses with greater intensity deliver more thermal energy, resulting in elevated temperatures and more extensive thermal wave penetration into the medium. According to the G-N II theory, which incorporates thermal wave propagation at finite speeds while precluding energy dissipation, heat transfer does not occur instantaneously—unlike the classical Fourier’s law prediction. Instead, it moves in the form of undamped thermal waves, highlighting a fundamental improvement over traditional diffusion-based models. Figure 2b shows the dimensionless displacement field $\tilde{u}$ along the same spatial domain $\tilde{x}$, under the influence of laser pulses with the same intensities. When a material is exposed to laser-induced heating, a rapid and localized rise in temperature occurs, leading to thermal expansion, which in turn gives rise to mechanical displacement within the medium. This displacement is a direct result of the thermoelastic coupling governed by the G-N II theory, which models finite-speed, non-dissipative thermal wave propagation. Several important physical effects are evident in this context. As the laser pulse intensity increases, the system absorbs more thermal energy, and this heightened thermal input leads to greater mechanical deformation. Notably, the displacement magnitude becomes more negative, implying either enhanced material contraction or a shift in the material’s equilibrium configuration due to the internal stresses induced by steep temperature gradients. Moreover, the spatial variation of displacement is clearly non-uniform across the medium, revealing that the mechanical behavior is highly sensitive to the spatial distribution of temperature. This non-uniformity stems from the nature of thermal wave propagation in the G-N II framework, where heat travels as a finite-speed wave, rather than diffusing instantly throughout the material. As a result, temperature gradients become steep and localized, leading to complex deformation patterns. Figure 3 depicts the stress tensor for Eq. (35) with the specified parameters  $g_2=4,\hspace{0.2cm}g_4=2,\hspace{0.2cm}\xi =-0.7,\hspace{0.2cm}b_1=\tilde{\textit{t}}, \text {and} \ \hspace{0.2cm}b _2=\tilde{\textit{t}}$. As $I_{0}$ increases, the stress field intensifies, showing how stronger thermal gradients lead to higher internal forces. The stress is positive and localized, indicating tensile behavior due to thermal expansion at early heating stages. This stress arises directly from the coupling between the thermal field and elastic response a key feature in thermoelasticity. Figure 4 depicts the dark soliton solutions from Eqs. (48), and (49) with the specified parameters  $g_2=15.9,\hspace{0.2cm}g_4=2,\hspace{0.2cm}\xi =-1.4,\hspace{0.2cm}b_1=\sqrt{\tilde{\textit{t}}}, \text {and} \ \hspace{0.2cm}b _2=1$. Figure (4a) illustrates that increasing the laser pulse intensity $I_{0}$ results in higher peak temperature values, indicating that more powerful laser pulses impart greater thermal energy into the material. The gradual, wave-like progression of the temperature profiles demonstrates the behavior of thermal waves rather than the instant spread expected from classical diffusion. In alignment with the G–N II theory, heat is transmitted through the medium at a finite speed without energy dissipation, maintaining the form of the wave over time. This behavior confirms that the laser pulse influences not only the magnitude of heating but also the rate and depth of thermal energy penetration into the material. Figure (4b) clarifies how mechanical displacement varies spatially under different $I_{0}$ values at the same time. Displacement increases significantly with rising laser intensity, indicating substantial thermoelastic deformation. The variation in displacement demonstrates that thermal gradients induce elastic expansion, as temperature changes generate stress through thermal expansion. Under G-N II theory, this behavior reflects a non-dissipative mechanical response to a structured thermal wave. The non-uniform displacement is tightly coupled to the localized heating induced by the laser, and the propagation of thermal waves affects how quickly the deformation spreads. Figure 5 depicts the stress tensor for (50) with the specified parameters  $g_2=15.9,\hspace{0.2cm}g_4=2,\hspace{0.2cm}\xi =-1.4,\hspace{0.2cm}b_1=\sqrt{\tilde{\textit{t}}}, \text {and} \ \hspace{0.2cm}b _2=1$. It is evident that the length of the laser pulse ($I_{0}$) plays a crucial role in determining both the magnitude and spatial distribution of stress within the material, where thermal and mechanical effects propagate as coupled waves under the G–N II framework. Shorter pulses amplify stresses but limit spatial spread, while longer pulses reduce peak stresses but broaden the impact, critical for laser-machining or material damage prediction.

## Conclusion

This study rigorously explores the derivation of precise wave solutions in nonlinear thermoelastic systems, with a focus on the significant influence of laser pulse effects. By employing advanced analytical strategies—most notably the MEDA method, a diverse range of explicit solutions has been systematically derived from the fundamental field equations governing the coupled thermo-mechanical interactions in temperature-sensitive media. These derived solutions provide critical insight into the complex interplay between thermally induced processes and mechanical responses, particularly in scenarios where laser pulse input and temperature-dependent material behavior play a pivotal role. The findings clearly indicate that the laser pulse significantly alters the nature of wave propagation within thermoelastic materials, leading to marked changes in dynamic behavior, including variations in stress distribution and displacement responses under varying external influences. The integration of temperature-dependent material characteristics into the theoretical framework enhances the model’s fidelity, enabling a more authentic and detailed representation of real-world thermoelastic responses. As a result, the predictive capacity of the G-N II thermoelastic theory is notably strengthened for applications involving high-temperature and thermally active environments. Furthermore, the obtained solutions encompass a broad array of waveforms, underscoring the adaptability and strength of the MEDA technique in effectively managing the inherent nonlinearities that often challenge traditional analytical approaches. The method’s flexibility allows it to extend and generalize classical methodologies, thereby offering deeper theoretical clarity on wave behavior in complex, coupled media. The theoretical advancements presented herein bear direct relevance to various practical engineering and scientific domains, such as the design of thermal shielding systems, the modeling of subsurface thermal phenomena in planetary exploration, and the analysis of thermo-mechanical performance in advanced structural materials subjected to extreme thermal loads. Ultimately, the versatility of these exact solutions renders them exceptionally useful for tailoring analyses across a wide spectrum of materials and loading configurations, making the present study a substantial contribution to the ongoing development of nonlinear thermoelastic wave theory under laser-driven and temperature-sensitive conditions.

## Data Availability

The datasets used and/or analyzed during the current study are available from the corresponding author upon reasonable request

## List of symbols

*u*: Component of the displacement vector in the medium
*k*: Material-specific constant in the constitutive equations
$\tau _{xx}$: Normal stress component along the *x*-axis
$\lambda$, $\mu$: Lamé’s first and second constants representing elastic properties
$\alpha _{t}$: Coefficient of linear thermal expansion; $\beta = (2\mu + 3\lambda ) \alpha _{t}$ defines thermoelastic coupling
$T_{0}$: Ambient reference temperature (undeformed state)
$\rho$: Mass density of the material
$\theta$: Temperature variation relative to $T_{0}$ ($\theta = T - T_{0}$)
$c_{e}$: Specific heat capacity at constant strain
*t*: Temporal variable
*Q*: Volumetric heat flux generated by the laser pulse
*r*: Gaussian beam radius characterizing laser spatial distribution
$\gamma$: Optical penetration depth for laser energy absorption
$t_{0}$: Characteristic time for laser pulse duration
$I_{0}$: Peak intensity of the absorbed laser energy per unit area
